# AhR Activation Transcriptionally Induces Anti-Microbial Peptide Alpha-Defensin 1 Leading to Reversal of Gut Microbiota Dysbiosis and Colitis

**DOI:** 10.1080/19490976.2025.2460538

**Published:** 2025-02-02

**Authors:** Manikandan Palrasu, Khadija Kakar, Amarnath Marudamuthu, Hamida Hamida, Shruthi Thada, Yin Zhong, Shanieka Staley, Philip Brandon Busbee, Jie Li, Monica Garcia-Buitrago, Mitzi Nagarkatti, Prakash Nagarkatti

**Affiliations:** a Department of Pathology, Microbiology and Immunology, University of South Carolina School of Medicine, Columbia, South Carolina, USA; b Department of Chemistry and Biochemistry, University of South Carolina, Columbia, South Carolina, USA; c Department of Pathology, University of Miami Miller School of Medicine, Miami, Florida, USA

**Keywords:** The aryl hydrocarbon receptor, inflammatory bowel disease, α-defensin 1, colitis, crohn’s disease, microbiome

## Abstract

Alpha-defensin 1 is a small antimicrobial peptide that acts as the first line of defense against pathogens. It is induced following microbial cues and inflammatory signals in neutrophils and Paneth cells in the small intestine, which suggests that it plays a role in microbial homeostasis in the gut. The gut microbial products also serve as ligands for the aryl hydrocarbon receptor (AhR), an environmental sensor. In the current study, we investigated if there is any crosstalk between AhR and alpha-defensin 1. Interestingly, we found a positive correlation between AhR and alpha-defensin 1 protein levels in ileal tissues from active Crohn’s’ (CD) patients and epithelial cells (IECs) from multiple models of murine colitis. *In vitro* downregulation of AhR led to inhibition of α-defensin 1, while activation of AhR induced α-defensin 1 in IECs. AhR directly targeted the dioxin response element 3 (DRE3) region on the α-defensin 1 promoter in IECs. AhR-mediated induction of α-defensin 1 in colitis mice reversed the gut microbial dysbiosis and alleviated colitis. Our data identify a novel signaling pathway in which AhR acts as a transcription factor for α-defensin 1, leading to regulation of homeostasis between gut microbiota, intestinal mucosa, and mucosal immunity.

## SUMMARY

- IBD patients and murine models of colitis had lower expressions of AhR and α-defensin 1.
- AhR activation on IECs induces the expression of α-defensin 1 by targeting the DRE sites of its promoter.
- α-defensin 1, in turn, reverses gut dysbiosis seen during colitis, and attenuates colonic inflammation and colitis.
- Targeting AhR-α-defensin 1 signaling pathway can serve as a therapeutic modality to treat dysbiosis and colitis as well as prevent other GI disorders including cancer.

## Introduction

Inflammatory bowel disease (IBD), a chronic inflammatory disease of the gastrointestinal tract, is considered the principal cause of colon cancer, which accounts for 10–15% of all IBD-related deaths.^1,2^ IBD is comprised of two main conditions: Crohn’s disease (CD) and ulcerative colitis (UC). UC is associated with inflammation and damage to the epithelial surface of the colon and rectum. However, CD promotes inflammation throughout the entire gastrointestinal system and damages it.^3,4^ A complex interaction of microbial, environmental, and host genetic factors trigger chronic inflammation and colitis,^1^ however, the mechanistic role of chronic inflammation in the pathogenesis of IBD and colon cancer is not well understood.

The aryl hydrocarbon receptor (AhR), a ligand-dependent transcription factor, plays a crucial role in maintaining gut health and protection against intestinal inflammation.^5^ The loss of the AhR was shown to enhance colitis development by dysregulating immune homeostasis, downregulating proinflammatory mediators, inhibiting epithelial barrier function, and modulating the common oncogenic signaling pathways, which includes FOXM1 and downstream genes, SOCS3-IL22-induced STAT3 activation, and Wnt signaling.^5–10^ Indeed, AhR activation was shown to reverse the aforementioned mechanisms, thus preventing UC.^5–10^ Activation of AhR by specific bacterial species and their metabolites has been found to alleviate symptoms in animal models of colitis and ulcerative colitis patients.^6,11^ Nevertheless, little is known about the role of AhR in regulating intestinal epithelial cell functions in gut inflammatory conditions.

Mouse α-defensins, or cryptdins, are antimicrobial peptides (AMPs) that protect the host from infections and are generated by Paneth cells in the intestinal Lieberkühn’s crypt.^12–14^ The mouse small intestine has yielded more than 20 cryptdin mRNAs thus far, of which the first six (Crp1 through Crp6) have been extracted and described at the peptide level.^15,16^ Human α-defensins are divided into myeloid defensins or human neutrophil peptides (HNPs) 1 to 4 and human (enteric) defensins (HDs) 5 and 6.^17–20^ As a result of activated neutrophil degranulation following holocrine secretion and neutrophil infiltration during inflammation, HNPs and cryptdins are secreted into the extracellular environment. Granules carrying HNPs are often targeted for fusion with phagolysosomes, where high HNP concentrations kill phagocytosed bacteria.^21^ HD5 and HD6 are constitutively expressed in Paneth cells at the bottom of a small intestinal crypt.^18^

Many studies reported the antimicrobial, antiviral, and antitoxic properties of α-defensins .^15–17,19,20^ However, little is currently known about how α-defensin 1 regulates microbial dysbiosis and innate immune responses in colonic epithelial cells. Antimicrobial peptides including α-defensin 1 and 5, protect against pathogens, influence the commensal microbiota, and regulate intestinal homeostasis.^22^ α-Defensin 1 has been shown to kill both Gram-negative and Gram-positive bacteria by membrane permeabilization.^23,24^ Equine α-defensin 1, which is only produced in Paneth cells, demonstrated antimicrobial efficacy against a wide range of microbes.^23^ Biopsies from adenomatous polyps showed α-defensin 1 expression accompanied by a significant relative reduction of mucosa adherent bacteria compared with normal tissue.^25^ In gut mucus, Meyer-Hoffert et al. discovered a variety of antimicrobial peptides including α-defensin 1 that demonstrated potent, contact-dependent antibacterial action against both beneficial and harmful microorganisms.^26^ In this study, we investigated how AhR activation affects gut microbiota and colonic inflammation by inducing α-defensin 1. Our analysis found that decreased expression of AhR protein and α-defensin 1 was found in ileal tissue from CD patients, and intestinal epithelial cells (IECs) from various colitis mice modes. Our results provide the first evidence that AhR activation transcriptionally induces the α-defensin 1 expression through binding to its DREs, leading to reversal of microbial dysbiosis and suppression of colitis. Downregulation of AhR may also affect host-bacteria interactions and tumorigenesis in the gut.

## Results

### Correlation between the expression of AhR and α-defensin 1 in CD patients

**Figure 1.**
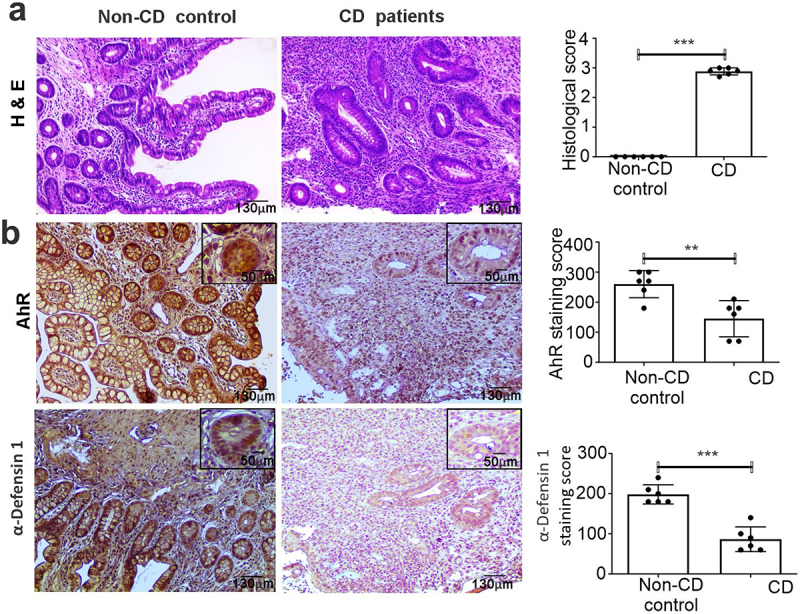
Expression of AhR and α-defensin 1 in ileal biopsies collected from active CD patients and non-CD control subjects (12 patients; 6/group). (a) H& E staining in active CD patients and non-CD control subjects. (b) AhR and α-defensin 1 proteins were analyzed by IHC staining in active CD patients and non-CD control subjects. Magnified views of AhR and α-defensin 1 expressions were depicted in insets, ×40. Scale bars: 50 μm. The graph panel shows histopathological and IHC scores (*n* = 12; 6 samples/group). Data were analyzed using unpaired 2-tailed t-test; Data are displayed as mean ± SD. **p < 0.01, ***p < 0.001.

To investigate the role of AhR in IECs under inflammatory conditions, we analyzed AhR protein expression by immunohistochemistry (IHC) in ileal tissue from active CD patients compared with the normal controls. Ileal biopsies from CD patients revealed severe active ileitis featuring irregular villous architecture and the discontinuous inflammatory infiltrates of cells in the lamina propria or in the epithelium (Figure 1a). Normal terminal ileal mucosa characterized by normal crypt architecture, lamina propria, and epithelium with no significant immune cell infiltrations was observed in ileal biopsies from non-CD controls (Figure 1a). IHC analysis found that AhR expression was downregulated in ileal tissue from active CD patients compared with the normal controls (Figure 1b). Our preliminary in silico analysis found that there are three AhR-binding DREs located ~9 kb upstream of the transcription start site (TSS) of α-defensin 1. We, thus, hypothesized that AhR may be involved in the regulation of α-defensin 1. To that end, we screened the expression of α-defensin 1 in ileal tissue from active CD patients and found that the α-defensin 1 expression was significantly downregulated when compared to the controls. Thus, there was a correlation between AhR and α-defensin 1 expression in active CD patients compared with the normal controls (Figure 1b).

### *In vivo* and *in* *vitro* activation of AhR causes upregulation of α-defensin 1 in IECs

Our analysis also found a significant downregulation of AhR and α-defensin 1 in the murine mouse models. We used I3C, a well-characterized AhR ligand, to activate AhR in murine models of colitis.^6,27^ We used three models of colitis: TNBS, anti-CD40 Abs, or DSS. For each colitis model, the animals were randomized into experimental and control groups and divided into four groups. These included 1) naïve (shown as control in the figures), 2) TNBS-/Anti-CD40/DSS+Vehicle (represented as TNBS-/Anti-CD40/DSS in the figures), 3) TNBS-/Anti-CD40/DSS+I3C, and 4) I3C alone. IECs were isolated from the ileal tissue of control and experimental animals and identified by EPCAM(+) staining using flow cytometry (Supplementary Figs. S1A-1C). We analyzed the expression of AhR and α-defensin 1 at mRNA and protein expression levels in IECs isolated from the ileum of control and experimental animals. Due to the fact that Paneth cells in the intestinal Lieberkühn’s crypt produce mouse α-defensins,^15,16^ we analyzed the expression of AhR and α-defensin 1 in IECs from control and mice with TNBS-, Anti-CD40-, and DSS-induced colitis.

**Figure 2.**
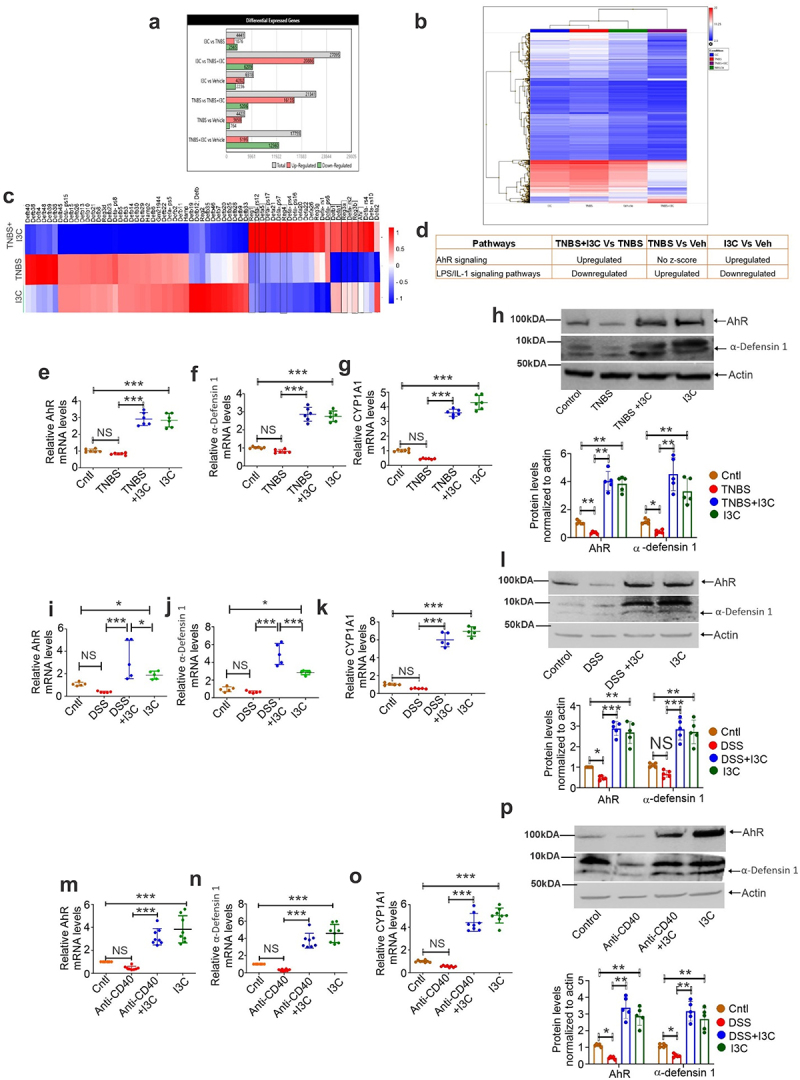
Expression of AhR and α-defensin 1 in IECs from control and TNBS-, anti-CD40- and DSS-induced colitis mouse models. (a-d) Whole transcript expression arrays of intestinal epithelial cells from TNBS-induced colitis mice treated with I3C (*n* = 3). (a) Differentially expressed genes in control and experimental animals. (b) Heatmap using Ward’s hierarchical clustering showing two-fold variations in many genes among different groups. (c) Heatmap depicting dysregulated antimicrobial peptides expressed as fold changes when compared to vehicle alone (d) Ingenuity pathway analysis of microarray data demonstrate that I3C activates AhR signaling pathways and represses LPS/IL-1 signaling. (e-g) Real-time PCR analysis for AhR (e), α-defensin 1 (f) and CYP1A1 (g) was performed on intestinal epithelial cells isolated from control and TNBS-induced colitis mice model (*n* = 6). (H) The same as e and f, but AhR and α-defensin 1 protein expression were analyzed (*n* = 5). (i-k) the same as e-g but, Real-time PCR analysis for AhR (i) α-defensin 1 (j) and CYP1A1 (k) was performed on intestinal epithelial cells isolated from control and DSS-induced colitis mouse model (*n* = 5). (l) The same as i and j, but AhR and α-defensin 1 protein expression were analyzed (*n* = 5). (m-o) the same as e-g but, Real-time PCR analysis for AhR (m) α-defensin 1 (n) and CYP1A1 (o) was performed on intestinal epithelial cells isolated from control and anti-CD40-induced colitis mice model (*n* = 8). (p) The same as m and n, but AhR and α-defensin 1 protein expression were analyzed (*n* = 5). Bottom panel for h, l and p represents densitometry analysis of AhR and α-defensin 1 protein expression. Data are shown as mean ± SEM, and significance was determined using 1-way ANOVA and Tukey’s multiple comparisons test; *p < 0.05; ***p < 0.001, NS=Not significant.

Whole-transcript expression array analysis of intestinal epithelial cells from TNBS-induced colitis mice treated with vehicle or I3C demonstrated several differentially expressed genes (Figure 2a). Heatmaps using Ward’s hierarchical clustering showed two-fold variations in many genes among different groups (Figure 2b). Figure 2c depicts a heatmap of dysregulated antimicrobial peptides (AMPs) represented as fold change in TNBS, TNBS+I3C, and I3C alone as compared to Vehicle alone. Downregulation of AhR expression in colitis mice was associated with downregulation of various antimicrobial peptides such as α-defensin 1, β-defensin1, reg1, reg4, reg3a, and reg3b. However, treatment with I3C, an AhR ligand, increased the expression of AhR and AMPs such as α-defensin 1 (Figure 2c). Ingenuity pathway analysis of microarray data demonstrates that I3C activates AhR signaling pathways and represses LPS/IL-1 signaling (Figure 2d). In addition, mRNA and protein expression of AhR and α-defensin 1 was assessed in IECs from control and mice with TNBS- (Figures 2e-h), DSS- (Figures 2i-l) and Anti-CD40 (Figures 2m-p)-induced colitis using real-time PCR and western blot analysis, respectively. However, unlike AhR and α-defensin 1 mRNA expression (Figures 2e,f,i,j,m and n) which was downregulated but not significantly in colitis mice compared to control, protein expression of AhR and α-defensin 1 was significantly downregulated in colitis mice than that of controls (Figures 2h,l,p). Interestingly, activation of AhR using I3C treatment increased the expression of α-defensin 1 at mRNA and protein levels in IECs (Figures 2e-p). We confirmed the AhR activation by assessing mRNA levels of the AhR target gene CYP1A1, which increased significantly in colitis mice treated with I3C (Figures 2g,k and o). Similar results were observed with 2,3,7,8-tetrachlorodibenzo-p-dioxin (TCDD), a potent activator of AhR in IECs from DSS- (Supplementary Figs. S1e, 2a-2d) and Anti-CD40 (Supplementary Figs. S1d, 2e-2 h)-induced colitis models. Our findings suggested that AhR expression was positively associated with the expression of α-defensin 1 in the murine colitis mice models.

**Figure 3.**
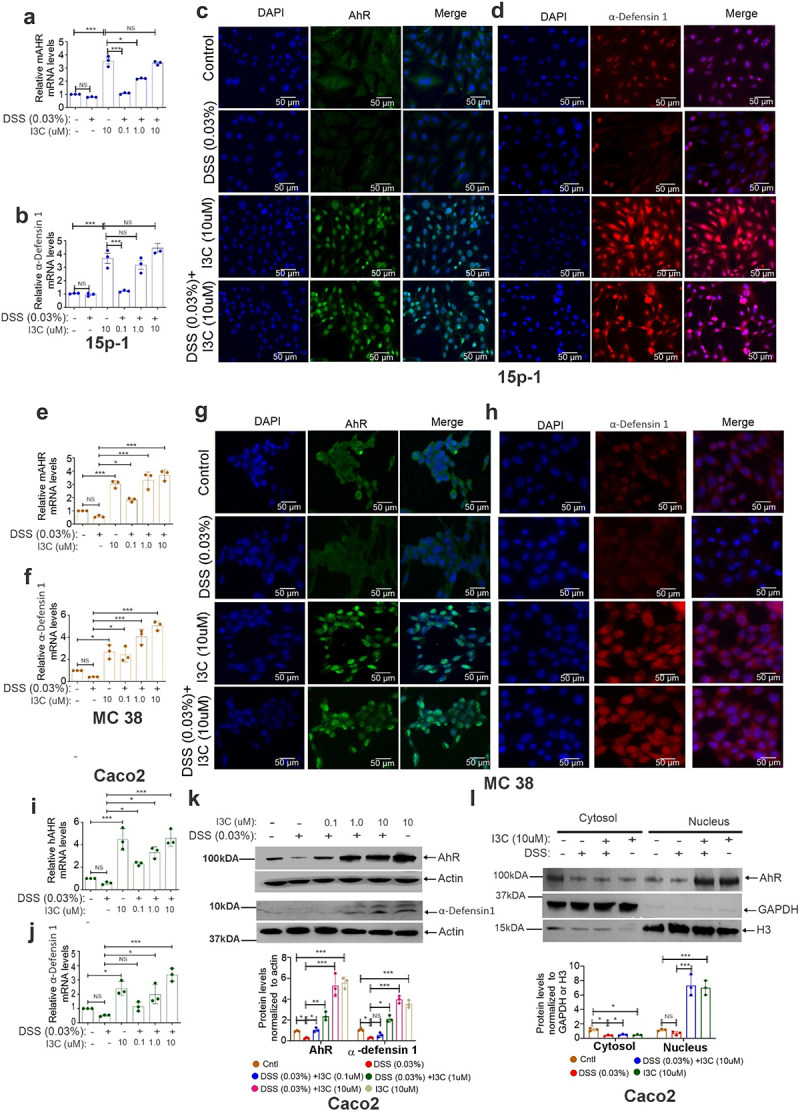
AhR activation enhances α-defensin 1 in epithelial cells *in vitro*. Epithelial cells indicated in figures were pretreated with I3C at indicated concentrations and then treated with DSS (0.03%) for an additional 16 hours. (a,b) the mRNA expression of AhR (a) and α-defensin 1 in 15p-1 cells (b) was analyzed by real-time PCR (*n* = 3). (c,d) AhR (c) and α-defensin 1 (d) protein expression was analyzed by immunofluorescence staining in 15p-1 cells (*n* = 3). Quantification of immunofluorescence staining using ImageJ was represented in supplemental figures 3C. (E,F) the same as (a,b), but AhR (e) and α-defensin 1(f) mRNA expression was analyzed in MC38 cells. (g,h) the same as (c,d) but AhR (g) and α-defensin 1(h) protein expression was analyzed by immunofluorescence staining in MC38 cells. Quantification of immunofluorescence staining was represented in supplemental figure 3D. (i,j) the same as (a,b), but AhR (i) and α-defensin 1 (j) mRNA expression was analyzed in Caco2 cells. (k) AhR and α-defensin 1 protein expression was analyzed by western blot in Caco2 cells (*n* = 3). (l) The same as k, but nuclear and cytosolic fractions were isolated and analyzed for the localization of AhR (*n* = 3) by Western blot. Data are shown as mean ± SEM, and significance was determined using 1-way ANOVA and Tukey’s multiple comparisons test; *p < 0.05; ***p < 0.001. NS=Not significant.

To explore how AhR activation affects α-defensin 1 expression in a more controlled environment, we employed additional *in vitro* experiments with murine adenocarcinoma cells, MC38, and human colon carcinoma, Caco2 cells. We also used murine Sertoli epithelial cells (15p-1), which are known to express α-defensin 1,^28^ to delineate the mechanism of the AhR-mediated regulation of α-defensin 1. We pretreated these epithelial cells with I3C (0.1, 1 and 10 µM) for 1 hour, and then treated them with DSS (0.03%) for another 16 hours. After 16 hours, we analyzed the mRNA and protein expression of AhR and α-defensin 1 by real-time PCR and western blot analysis, respectively. Protein expression of AhR and α-defensin 1 was significantly downregulated in DSS-treated 15p-1 cells (Figures 3c,d and Supplementary Figs. S3a and 3b) compared to control, in contrast to AhR and α-defensin 1 mRNA expression (Figures 3a,b), which was downregulated in DSS treated 15p-1 cells but not significantly. However, I3C treatment increased the expression of AhR and α-defensin 1 at mRNA (Figures 3a,b) and protein (Figures 3c,d and Supplementary Figs. S3a and 3b) levels in 15p-1 Sertoli epithelial cells in a dose-dependent manner. In particular, the nuclear accumulation of AhR protein expression in 15p-1 cells (Figure 3c), correlated with the upregulation of α-defensin 1 protein expression (Figure 3d) upon I3C treatment. Similar results were observed in MC38 (3e-h and Supplementary Figs. S3c and 3d) and Caco2 (Figures 3i-l). Like the *in vivo* colitis studies, our findings in Sertoli epithelial cells and IECs demonstrated that AhR activation leads to upregulation of α-defensin 1. It was interesting to note that I3C alone was also able to increase the induction α-defensin 1 in these cells.

### AhR activation transcriptionally regulates α-defensin 1 in IECs in vitro and in vivo

**Figure 4.**
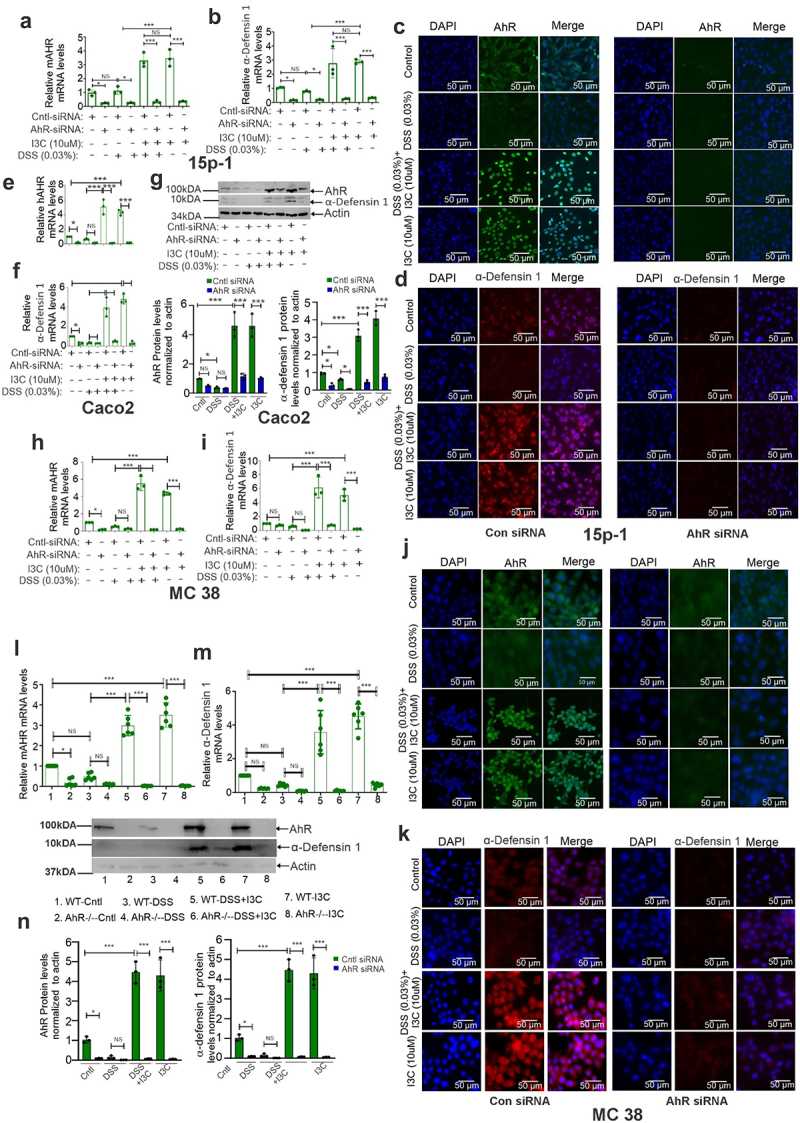
Downregulation of AhR leads to inhibition of α-defensin 1 induction in epithelial cells *in vitro* and *in vivo* (a-d) 15p-1 cells transiently transfected with AhR siRNA or control siRNA were pretreated with I3C, at indicated concentrations, and then treated with DSS (0.03%) for an additional 16 hours. AhR (a) and α-defensin 1 (b) mRNA expression was analyzed by real-time PCR. (c, d) AhR (c) and α-defensin 1 (d) protein expression was analyzed by immunofluorescence staining (*n* = 3). Quantification of immunofluorescence staining was represented in supplemental figure 4b (*n* = 3). (e, f) the same as (a,b), but the Caco2 cells were used. (g) AhR and α-defensin 1 protein expression was analyzed in Caco2 cells by western blotting (*n* = 3). (h, i) the same as (a, b), but the MC38 cells were used. (j, k) the same as (c, d), but the MC38 cells were used. Quantification of immunofluorescence staining was represented in supplemental figure 4d (*n* = 3). (l-n) IECs were isolated from IEC-specific conditional AhR knockout mice (AhRΔIEC), and wild-type (WT) mice having colitis induced by DSS as indicated in figures and analyzed the mRNA expression of AhR (l), and α-defensin 1 (m) by real time-PCR. (k) The same as (l, m), but the protein expression of AhR and α-defensin 1 was analyzed by Western blot (*n* = 6). Data are shown as mean ± SEM, and significance was determined using 1-way ANOVA and Tukey’s multiple comparisons test; **p < 0.05; **p < 0.01; ***p < 0.001. NS=Not significant.

To investigate the impact of AhR in the regulation of α-defensin 1 in IECs, its expression was downregulated with AhR antagonists or AhR-specific siRNA, in 15p-1 cells. Initially, we treated 15p-1 cells with I3C (10 µM) after being pretreated for 2 hours with AhR antagonists, α-Naphthoflavone (NP; 5 µM) or CH223191 (CH; 10 µM). AhR and α-defensin 1 mRNA and protein expression were examined using real-time PCR and immunofluorescence staining. Our analysis found that AhR and α-defensin 1 expression at mRNA (Supplementary Figs. S3e and 3f) and protein levels (Supplementary Figs. S3g and 3 h) were induced by I3C treatment; however, in cultures containing antagonists, the induction of α-defensin 1 was diminished (Supplementary Figs. S3e-3 h). We also downregulated AhR using AhR-specific siRNA in 15p-1 cells that were then pretreated with I3C (10 µM) followed by DSS (0.03%) treatment for additional 16 hours and analyzed for the expression of AhR and α-defensin 1 at mRNA (Figures 4a,b) and protein (Supplementary SFig4a; Figures 4c,d; Supplementary Fig S4b) levels. Our studies revealed that downregulation of AhR reproducibly inhibited the induction of α-defensin 1 at mRNA and protein levels upon I3C treatment (Figures 4a-d; Supplementary Figs. S3g, 3h, 4a, and 4b). On the other hand, in 15p-cells transfected with control/control siRNA but not AhR-siRNA/antagonists, I3C treatment increased the activation and nuclear accumulation of AhR (Figures 4c,d; Supplementary Figs. S3g, 3h, and 4b), which was correlated with elevated expression of α-defensin 1 (Figures 4c,d; Supplementary Figs. S3g, 3h, and 4b). Similar results were also seen in Caco2 cells (Figures 4e-g) and MC38 cells (Figures 4h-k; Supplementary Figs. S4c, and 4d). We also analyzed the expression of α-defensin 1 in IECs from IEC-specific conditional AhR knockout mice (AhRΔIEC), and wild-type (WT) mice having colitis induced by DSS. Consistent with *in vitro* findings, our *in vivo* investigation revealed that whereas I3C treatment induced AhR and α-defensin 1 expression in wild-type (WT) mice (Figures 4l-n), it was unable to induce AhR and α-defensin 1 mRNA and protein expression in IEC-specific conditional AhR knockout mice (AhRΔIEC) (Figures 4l-n).

**Figure 5.**
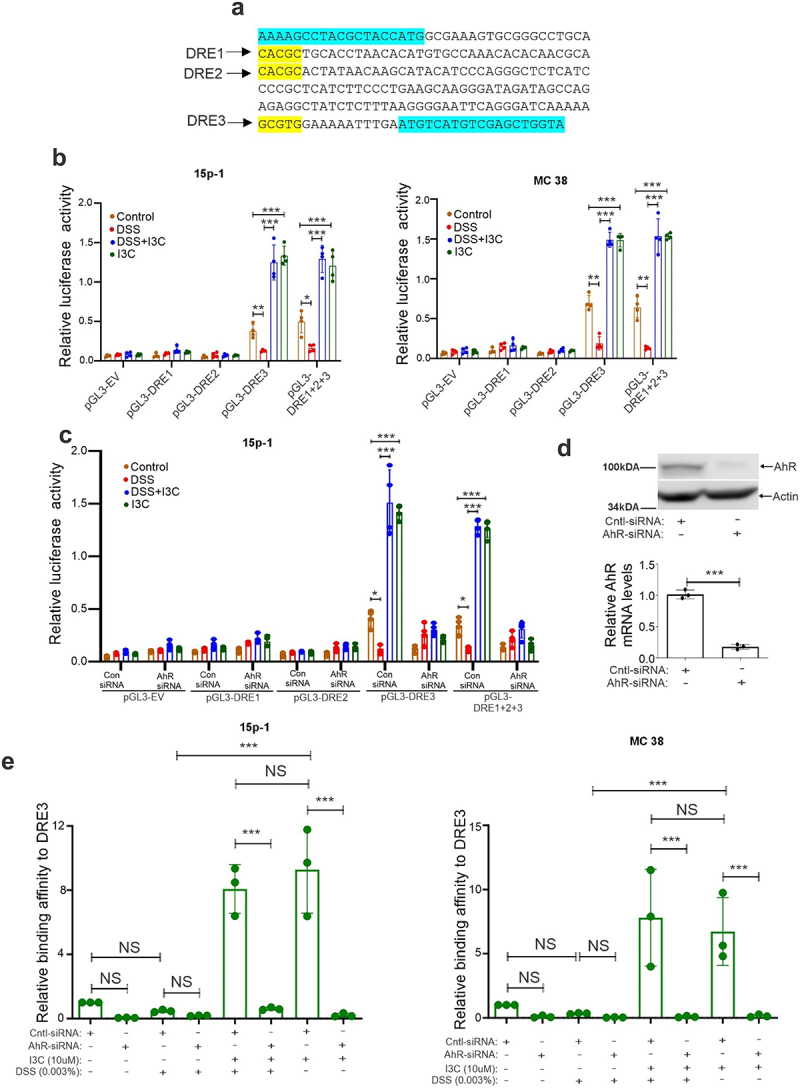
AhR activation promotes the expression of α-defensin 1 via transcriptional binding of AhR to a DRE3 on α-defensin 1 promoter. (a) Identification of three DREs in α-defensin 1 promoter by *in silico* analysis. (b) 15p-1 and MC38 cells were transfected with pGL3-DRE1, pGL3-DRE2, pGL3-DRE3, and pGL3-DRE1 + 2 + 3 luciferase reporter plasmid and then treated with I3C and DSS at indicated concentration for 16hrs and luciferase reporter assays were performed (*n* = 4). (c) Same as A and B, but MC38 cells transiently transfected with AhR siRNA or control siRNA were used (*n* = 4). (d) The transfection efficiency of the reporter assay for AhR siRNA was measured via western blotting analysis with antibodies against AhR (upper panel) and RT-PCR analysis of AhR mRNA expression (bottom panel). All luciferase assays were normalized for transfection efficiency by renilla reporter activity. The results shown are a representative of four independent experiments performed each time in triplicate. (e) ChIP assay using AhR antibody followed by qPCR applying primers covering DREs demonstrate that DRE3 region containing α-defensin 1 promoter fragment can be pulled down more by AhR antibodies in 15p-1 and MC38 transfected cells with control siRNA compared to corresponding AhR-siRNA transfected cells (*n* = 3). Data are shown as mean ± SEM, and significance was determined using 1-way ANOVA and Tukey’s multiple comparisons test; ***p < 0.001. NS=Not significant.

To define the association between AhR and an enhanced level of α-defensin 1 expression, we asked whether AhR plays a direct role in the regulation of α-defensin 1 mRNA expression. In that context, we found three AhR binding DREs at α-defensin 1 promoter (Figure 5a) using *in silico* analysis. Thus, we generated a luciferase reporter encompassing the α-defensin 1 promoter and termed it pGL3-DRE1, pGL3-DRE2, pGL3-DRE3, and pGL3-DRE1 + 2 + 3 depending on the DRE regions. These reporter plasmids were transfected into 15p-1 and MC38 cells and then treated with I3C (10 µM) and DSS (0.03%) for 16 hours (Figure 5b). Through the reporter assay, we found that AhR activation by I3C treatment showed higher luciferase activity when transfected with the pGL3-DRE3 alone and pGL3-DRE1 + 2 + 3 compared to the cells transfected with an empty vector or the reporter with other DRE regions (Figure 5b).

To validate further how AhR is involved in the regulation of α-defensin 1 luciferase activity, we co-transfected pGL3-DRE1, pGL3-DRE2, pGL3-DRE3, and pGL3-DRE1 + 2 + 3 plasmids together with AhR/control siRNA into 15p-1 cells (Figure 5d). Our analysis found that I3C treatment significantly increased protein expression of AhR (Figure 3c, Supplementary Figs. S3a, 3b) and induced α-defensin 1 luciferase activity in 15p-1 cells transfected with control siRNA (Figure 5c). On the contrary, I3C treatment in AhR downregulated cells (Figure 5d) inhibited α-defensin 1 promoter activity (Figure 5c). Similar results were observed in MC38 cells (Supplementary Figs. S4e, 4f).

To support the reporter activity observed, we conducted ChIP analysis on the binding potential of AhR protein to the DRE3 regions of α-defensin 1 promoter in 15p-1 and MC38 cells transfected with control and AhR-downregulated cells. We found that antibodies against AhR can also pull down a substantial amount of DRE3 in 15p-1 and MC38 cells transfected with control siRNA (Figure 5e) compared with the AhR-downregulated cells. Combined, these findings demonstrated that AhR activation by I3C can regulate α-defensin 1 mRNA expression through the association with a DRE3 region of α-defensin 1 promoter.

### AhR activation protects colitis and reverses microbial dysbiosis through α-defensin 1 induction *in* *vivo*

**Figure 6.**
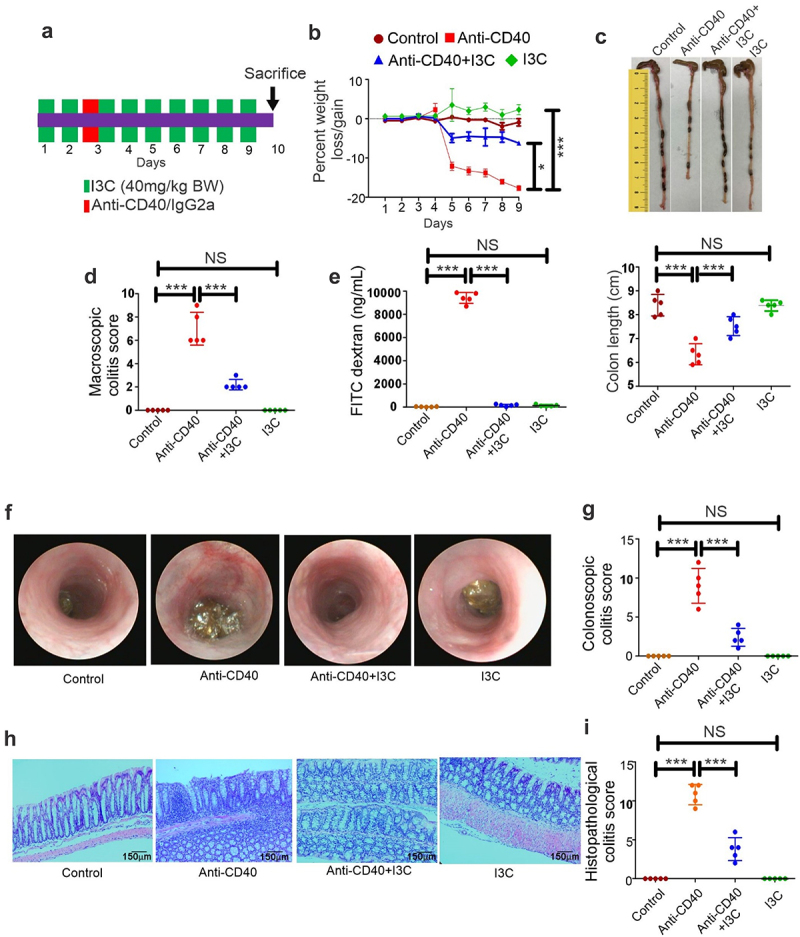
AhR activation induces α-defensin 1 and attenuates colitis *in vivo*. (a) Experimental design for anti-CD40-induced colitis mice model as described in Methods. (b –e) Percent weight loss (b), colon length (c), macroscopic score (d), FITC-dextran (e) as a measure of gut permeability and damage was measured and used as marker for colitis. (f) Representative colonoscopy images of the experimental and control animals (Left panel). (g) Graph depicting colonoscopy scores from experimental mice (Right panel) (*n* = 5). (h) Representative H&E stains of colons from experimental mice (Left panel). Scale bars: 140 μm (original magnification, ×10). (i) Graph depicting histopathological scores of H&E-stained colons from experimental mice (Right panel) (*n* = 5). Data are displayed as mean ± SD. Significance was determined using 1-way ANOVA and Tukey’s multiple comparisons test; ***p < 0.001, NS=Not significant.

Our data raised an important question about the potential biological impact of α-defensin 1 induction by AhR activation in colitis mice models. It has been reported that the loss of the AhR enhances colitis development, and AhR activation has been shown to prevent colitis.^6,29,30^ Our findings consistently found that AhR activation by I3C was positively correlated with the induction of α-defensin 1 in ileal epithelial cells from control and mice with TNBS- (Figures 2e,f and h), DSS- (Figures 2i,j,l) and anti-CD40 (Figures 2m,n and p)-induced mice models. We, therefore, analyzed the various clinical parameters in anti-CD40- (Figures 6a-i), DSS- (Supplementary Figs S5A–5I) and TNBS (Supplementary Figs S6a–6i)-induced colitis. Our analysis found that anti-CD40-induced colitis mice displaying decreased AhR-mediated α-defensin 1 induction exhibited increased body weight loss (Figure 6b), colon shortening (Figure 6c), macroscopic colitis score (Figure 6d), increased serum FITC-dextran (Figure 6e), a marker for gut permeability, increased colonic (Figure 6f,g) and histopathological colitis scores (Figures 6h,i) as a measure, based on marked crypt architecture damage, inflammatory cell infiltration and ulceration. However, α-defensin 1 which was induced by AhR activation by I3C maintained crypt development and normal colonic tissue architecture and attenuated colitis. Similar results were observed in DSS- (Supplementary Figs S5a-5i) and TNBS-colitis mice models (Supplementary Figs S6a-6i).

**Figure 7.**
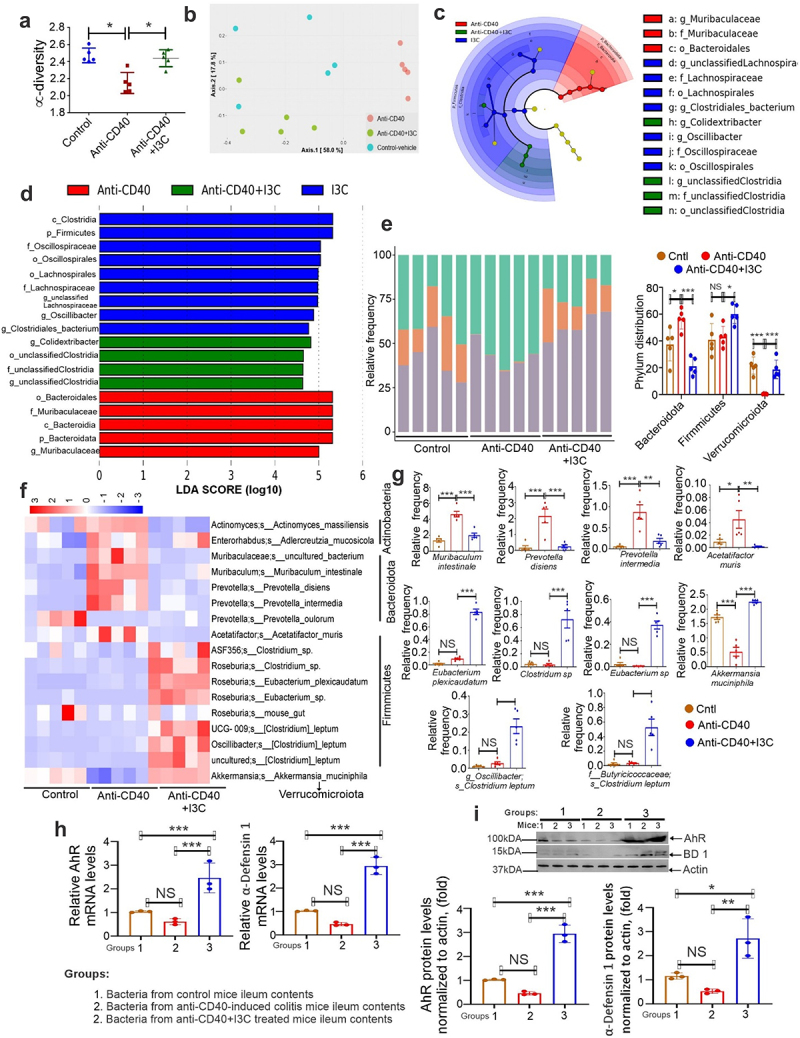
AhR-mediated induction of α-defensin 1 reverses microbial dysbiosis *in vivo*. 16S rRNA sequencing was performed from the ileal contents of control and anti-CD40-induced colitis mice. Sequenced reads were analyzed using Nephele to determine chao1 α diversity (a) and β diversity by PCA plot (b). (c) Cladogram (d) and LDA score depicting biomarkers among different groups. (e) A more detailed phylum distribution in control and experimental animals. The graph (right) panel shows the levels of Bacteroidota, Firmicutes and Verrucomicroiota phylum in control and colitis mice (*n* = 5). (f,g) Heatmap (f) depicts a more detailed species distribution and the graph panel (g) represents the levels of various species in control and experimental animals (*n* = 5). (h) The mRNA expression of AhR and α-defensin 1 in MC38 cells cocultured with the bacterial population from ileal contents of control, anti-CD40-induced colitis and anti-CD40+I3C treated mice for 24hrs. (i) The same as h, but we analyzed the protein expression of AhR and α-defensin 1 (*n* = 3). Data are shown as mean ± SD. Significance was determined using 1-way ANOVA and Tukey’s multiple comparisons test; *p < 0.05; ***p < 0.001, NS=Not significant.

Previous studies have demonstrated a significant contribution of microbiome dysbiosis to AhR dysregulation and colitis development.^6,9,11,31^ Because α-defensins are known to protect the gut from microbial colonization,^15,16^ we investigated how microbial dysbiosis in colitis mice was affected by AhR-mediated production of α-defensin 1. We analyzed microbiome composition in the ileal contents of control and anti-CD40 or DSS-induced colitis animals by using the 16S rRNA sequencing. Nephele was utilized to analyze the sequenced reads to determine the α (Figure 7a; Supplementary Figs. S7a) and β diversity (Figure 7b; Supplementary Figs. S7b) of chao1 using PCA plot. Compared to controls, mice with anti-CD40 or DSS-induced colitis showed a substantial decrease in chao1 α diversity (Figure7a; Supplementary Figs. S7a). On the other hand, chao1 α diversity in mice with colitis increased upon AhR activation by I3C treatment (Figure7a; Supplementary Figs. S7a). Moreover, animals with colitis demonstrated a significant variation in β diversity from the treatment (Anti-CD40/DSS + I3C) and control groups in their ileal samples (Figure 7b; Supplementary Figs. S7b). Cladogram (Figure 7c; Supplementary Figs. S7c, 7d) and LDA score (Figure 7d; Supplementary Figs. S7e, 7f) generated using LEfSe depicted differentially enriched microbial taxa from the phylum to the genus level between control, anti-CD40 or DSS-induced colitis. Our analysis found that anti-CD40 or DSS-induced colitis mice had enrichment of Bacteroidia phylum members, which exacerbates intestinal inflammation by producing LPS (Figure 7e; Supplementary Figs. S7g). However, the numerous Firmicutes phylum members that maintain colon health by generating butyrate and other AhR ligands were reduced in colitis mice (Figure 7e; Supplementary Figs. S7g). Furthermore, compared to control animals, the abundance of the Verrucomicrobiota phylum decreased steadily in mice with anti-CD40-induced colitis (Figure 7e), whereas the Verrucomicroiota phylum did not change significantly in mice with DSS-induced colitis (Supplementary Fig. S7g). AhR activation by I3C treatment enhanced the many members of the *Firmicutes* as well as *Verrucomicroiota* phylum and decreased the members of *Bacteroidia* phylum in colitis mice (Figure 7e; Supplementary Fig. S7g). At species levels, we observed differences in microbiota composition in control and colitis mice, summarized in heatmaps (Figure 7f; Supplementary Fig. S7h) and bar plots (Figure 7g; Supplementary Fig. S7i). Our analysis found that, at the species level, Muribaculum intestinale, Prevotella disiens, Prevotella intermedia from Bacteroidia phyum members and Acetatifactor muris from Firmicutes were significantly increased in colitis mice, compared to controls. In contrast, control or anti-CD40-induced colitis mice treated with I3C showed lower levels of Muribaculum intestinale, Prevotella disiens, Prevotella intermedia and Acetatifactor muris (Figures 7f,g). Interestingly, Firmicutes phylum members such as Eubacterium plexicaudatum, Clostridium sp, and Eubacterium sp from genus Roseburia, Akkermansia muciniphila from Verrucomicroiota phylum, Clostridium leptum from genus Oscillibacter and Clostridium leptum from family Butyricicoccaceae showed a significant increase in anti-CD40+I3C treated mice when compared to anti-CD40+Vehicle groups (Figures 7f,g). Similar results were seen in ileal contents from the DSS-induced colitis mouse models (Supplementary Figs. S7a-7i). However, *Alistipes onderdonkii* species from Firmicutes phylum increased in DSS+I3C treated mice when compared to DSS+Vehicle groups (Supplementary Figs S7e and 7i). Together, these data suggested that treatment with I3C, particularly in colitis-induced conditions, significantly altered the gut microbiome composition.

Next, we investigated the impact of ileal microbiota on AhR and BD-1 expression in CECs. We cocultured a bacterial population from the ileal contents of normal, anti-CD40-induced colitis, and anti-CD40+I3C treated mice with CECs MC38 for 24 hours and examined AhR and BD1 mRNA (Figure 7h) and protein expression (Figures 7i). Our analysis found that microbiota population from anti-CD40+I3C treated mice significantly increased the expression of AhR and BD1 compared to cells cocultured with bacterial population from colitis mice (Figures 7h-i). Taken together, our findings suggested that AhR activation by I3C treatment in colitis mice reverses microbial dysbiosis and attenuates colitis.

## Discussion

Our studies provide the first evidence that AhR activation transactivates α-defensin 1 expression through binding to DRE regions on α-defensin 1 promoter. This results in the reversal of microbial dysbiosis and suppresses colonic inflammation and colitis. Herein, we report a decrease in antimicrobial peptide α-defensin 1 in the ileal mucosa of CD patients and ileal cells from multiple colitis murine models. We recapitulated these changes *in vitro* using DSS-treated Sertoli and IECs as an *in* *vitro* inflammatory model of the human intestinal epithelium. α-Defensins are generated by Paneth cells in the intestinal Lieberkühn’s crypt to defend the host intestine from virulent microbes.^12–14^ TNBS-induced colitis shows similarities to human Crohn’s disease that promotes inflammation throughout the entire gastrointestinal system including Paneth cells in the intestinal Lieberkühn’s crypt and damages it.^3,6^ Thus, we first analyzed the differential expression of various genes in IECs from control and TNBS-induced colitis mice using whole-transcript expression array analysis. Interestingly, our investigation demonstrated that AhR activation in TNBS-induced colitis was positively correlated with the expression of α-defensin 1. We consistently observed similar findings in DSS- and anti-CD40-induced colitis models. Furthermore, whole-transcript expression array analysis also revealed that AhR influences the expression of a variety of AMPs such as Reg1, Reg3a, Reg3b, and Reg4, with the majority of those AMPs induced via AhR-independent regulation because of the lack of expression of DREs on their promoters. Further research is underway to determine how AhR regulates these AMPs.

Although multiple studies reported that α-defensins 1–3 are expressed by neutrophils,^13–15^ immunohistochemical analysis revealed that surface enterocytes were strongly positive for α-defensins 1 in non-CD control patients. Western blot analysis in IECs from mice also showed a significant expression of α-defensins 1. Additionally, the Sertoli and intestinal epithelial cells were able to recapitulate α-defensin-1 expression *in vitro*. However, it’s unclear if α-defensin 1 expression in human non-CD tissue and IECs from mice occur on the epithelium or if enterocytes just absorb it from nearby neutrophils. Reduced Paneth cell defensin expression has been documented in ileal Crohn’s disease patients.^32^ Our findings align with the aforementioned finding indicating that decreased α-defensin 1 expression in human CD patients may impair mucosal host defenses and increase the risk of intestinal mucosal inflammation. This is evident from the IECs derived from various colitis mouse models.

AhR is expressed in epithelial and immune cells and acts as a transcription factor. It binds to dioxin response elements (DREs) in the nucleus and activates genes involved in immune response and homeostasis.^5^ Our findings on AhR are consistent with previous observations showing reduced AhR protein in active CD and UC patients.^33^ We also observed similar findings in IECs *in vivo* and *in vitro*. In particular, AhR activation by I3C or TCDD, well-characterized ligands for AhR, induced the mRNA and protein expression of α-defensin 1 in IECs from multiple colitis mouse models and various epithelial cells *in vitro*. Furthermore, by using in silico analysis, we identified conserved AhR binding DREs in the mouse α-defensin 1 promoter, which further encouraged us to investigate the transcriptional regulation of defensin 1 through AhR. Our analyses in AhR downregulated cells and IEC-specific conditional AhR knockout mice (AhRΔIEC)-having colitis induced by DSS demonstrated diminished ability to induce α-defensin 1 mRNA and protein expression upon I3C treatment. However, I3C treatment enhanced AhR activation followed by α-defensin 1 induction in wild-type colitis mice.

Consistent with *in vivo* results, downregulation of AhR inhibited the induction of α-defensin 1 and we were able to recapitulate α-defensin 1 induction upon AhR activation by I3C *in vitro* using Sertoli cells and IECs. We further substantiated that the α-defensin 1 was a target of AhR by detecting the high activity of a luciferase reporter containing the AhR binding DRE3 region of α-defensin 1 promoter in Sertoli cells and IECs. Moreover, ChIP and reporter assays demonstrated that AhR activation by I3C treatment could substantially upregulate α-defensin 1 mRNA through the AhR binding DRE3 region of α-defensin 1 promoter as compared to AhR downregulated cells, which showed a minimum effect on the regulation of α-defensin 1 expression. Our studies suggest that AhR may function as a post-transcriptional regulator of α-defensin 1 in normal individuals and that in CD patients, dysregulation in AhR and α-defensin 1 may initiate the inflammatory colitis process. While it has been demonstrated that activation of AhR inhibits colitis through many signaling pathways that involve immune cells,^6–8,11,30^ we discovered a new potential mechanism by which AhR can prevent colonic inflammation and colitis through AhR-mediated transcriptional induction of α-defensin 1 in IECs.

Our analysis consistently found a more robust decrease in AhR and α-defensin 1 protein levels in colitis vs control mice and cells treated vs untreated with DSS, when compared to the data generated using mRNA analysis. This suggested that there could be inhibition of the post-translational modifications. After activation of AhR and subsequent induction of genes that express DREs, a number of post-translational alterations can affect the expression of the targeted genes.^34^ These pathways include ubiquitination, phosphorylation, methylation, acetylation, glycosylation, and proteolytic cleavage. The downregulation of AhR has been shown to be ubiquitin-mediated involving the 26S proteasome pathway after the nuclear export of AhR.^35^ Thus, AhR degradation may also play a role in gene regulation. It is noteworthy that colitis has been shown to be associated with several posttranslational protein modifications such as phosphorylation, neddylation, hydroxylation, and cleavage of cytokine precursors by the inflammasome, which regulate the inflammation and colitis pathogenesis.^36^

AhR activation during colitis has also been shown to downregulate the mucosal immune response by modulating many other signaling pathways. AhR activation by dietary compounds such as I3C, xenobiotics such as TCDD, and microbial components such as tryptophan metabolites and SCFAs have been shown to promote anti-inflammatory pathways such as induction of IL-10, IL-22, prostaglandin E2, and Foxp3.^6,31^ We and others have shown that I3C treatment attenuates colonic inflammation and reveres microbial dysbiosis primarily through the induction of IL-22.^6,11,37^ Clinical findings demonstrating lower levels of gut microbiota-derived AhR ligands in colitis patients further establish the link between AhR ligands and IBD.^11^ Our analysis in this study demonstrates that AhR-dependent induction of α-defensin 1 in IECs is yet another potential mechanism by which AhR can circumvent microbial dysbiosis, inflammation, and colitis.

Commensal bacteria and the gut mucosa work together meticulously to maintain a delicate balance in a healthy state. Any disruption of this equilibrium can lead to bacterial invasion of the mucosa and increase the risk of developing chronic inflammation associated with IBD.^32,38^ The exact mechanisms, nevertheless, that connect intestinal microbes and host factors remain unclear. The terminal ileum is most abundant in microbiota and commonly affected areas during CD pathogenesis, among other parts of the small intestine.^39^ It contains Peyer’s patches, a group of lymphoid follicles in the mucus membrane that serve as important immunological regulators in the small intestine, allowing microorganisms to interact with immune cells.^39,40^ Antimicrobial peptides produced by host cells of the epithelial lining of the small intestine, such as α-defensins, act as a barrier against pathogens.^41^ Little study on the microbiota of the ileum has been conducted due to sample challenges and adverse milieu. A recent study discovered that transferring IBD-associated microbiotas to mice promotes intestinal inflammation and exaggerates colitis, implying a broader mechanism for microbial contribution to IBD pathogenesis.^42^ Our results show that α-defensin 1 deficiency in CD patients and various mouse models of colitis affect intestinal mucosa antimicrobial defenses and cause progressive alterations in surface and luminal bacteria composition.

Multiple studies demonstrate that IBD patients and mice with colitis exhibit significant increases in the abundance Bacteroidetes and a decrease in Firmicutes at the phylum level,^43–45^ and this imbalance may exacerbate intestinal inflammation. In the current study, the control and anti-CD40-induced colitis mice did not differ significantly in Firmicutes, but they did exhibit a decline in the Verrucomicrobiota phylum, which did not change significantly between control and DSS-induced colitis. Previous research has found that Verrucomicrobiota phylum members such as *Akkermansia muciniphila* decreased in ulcerative colitis patients and colitis animal models, which supports our findings.^46–48^ In a murine anti-CD40-induced colitis model, we found significant increase in Muribaculum intestinale, Prevotella disiens, Prevotella intermedia from Bacteroidia phylum members and Acetatifactor muris from Firmicutes, which correlated with intestinal mucin degradation and inflammation,^49–51^ whereas Firmicutes phylum members such as *Lactobacillus intestinalis*, *Eubacterium plexicaudatum*, *Clostridium sp*, and *Eubacterium* sp from genus Roseburia, *Clostridium leptum* from genus *Oscillibacter* and *Clostridium leptum* from family Butyricicoccaceae decreased during inflammation, correlating with a reduction in butyrate.^6,52–54^ We discovered that anti-CD40-induced colitis mice had a decrease in the abundance of *Akkermansia muciniphila*, which has been shown to degrade host mucin in various products such as short chain fatty acids to regulate host biological functions and immune homeostasis.^55,56^ Consistent with our findings, many studies reported that reduced abundance of *Akkermansia muciniphila* was associated with inflammatory bowel disease in both mouse and human studies.^47,55^ Reduced abundance of butyrate producing bacteria in colitis patients and chemically induced colitis facilitates the buildup of potentially harmful bacteria such as Bacteroidetes phylum members,^57,58^ as seen in colitis mice.

Interestingly, our findings are consistent with previous observations showing that AhR activation using specific ligands such as I3C significantly reduces many members of Bacteroidia phylum and increases Firmicutes and Virrucomicroiota in colitis mouse models.^6,46^ In particular, *Muribaculum intestinale*, *Prevotella disiens*, *Prevotella intermedia*, and *Acetatifactor muris* are among the numerous colitis-causing bacteria that may be controlled by AhR-dependent induction of α-defensin 1. This suggests that inhibiting these bacteria aids in the growth of numerous Firmicute and Virrucomicroiota bacterial species. It has been shown that equine α-defensin1 can kill a wide range of microbes,^23,24^ and increased expression of α-defensin 1 was associated with mucosa adherent bacteria in colonic adenoma mucosa.^25^ Our findings demonstrate that bacterial species from Firmicutes and Virrucomicroiota are increased when AhR is activated using I3C treatment, which is consistent with animal studies in which bacteria such as *Akkermansia muciniphila*, *Roseburia*, and other butyrate-producing bacteria were found to be anti-inflammatory, restoring and maintaining normal intestinal barrier integrity.^55,59,60^

Numerous investigations have confirmed that mucosal defensins play a crucial role in the pathogenesis of IBD due to their antibacterial activity, which aligns with our findings.^14,16,32,33,61^ Apart from altering the makeup of microorganisms, α-defensin 1 has the potential to regulate the actions of diverse immune cells. Conversely, colitis mice and IBD patients’ intestines may contain microorganisms that directly impact the production of α-defensin 1 through activation of AhR. Our results revealed that the microbiota population from colitis mice treated with I3C dramatically elevated AhR and α-defensin 1 expression. To support the aforementioned findings, a recent study found that the AhR/IL-22/Stat3 signaling pathway is used by commensal microbiota to modify antimicrobial peptides in the intestinal mucosa.^62^ Collectively, our research reveals that AhR activation via I3C therapy in colitis mice transcriptionally increases α-defensin 1, reversing microbial dysbiosis as demonstrated by an increase in Firmicutes and Virrucomicroiota bacteria and a decrease in gram-negative bacteria, ultimately suppressing colitis.

In summary, our studies demonstrate that AhR is an essential host factor that transcriptionally induces α-defensin 1 in IECs to regulate gut microorganisms, thereby reversing microbial dysbiosis and attenuating colitis. Our findings suggest that restoring the host–microbe balance at the intestinal mucosa via targeting AhR is an effective therapeutic approach to inhibit IBD and potentially IBD-associated colon cancer.

## Material and methods

### Human tissue samples

Human ileal tissue specimens from both non-CD controls and patients with severe CD were obtained from the University of Miami, Miami, and the Institutional Review Board of the University of Miami approved the protocols for human studies. AhR and α-defensin1 protein expression was assessed by immunohistochemistry (IHC) using Abs to AhR (Cat#sc-133088) from Santa Cruz Biotechnology (Santa Cruz, CA) and Abs to α-defensin1 (Cat# PA5-103122) from Invitrogen as previously described.^63^ The percentage of positive cells was multiplied by the intensity score, which was ranked as 0 (negative), 1 (weak), 2 (moderate), or 3 (strong), to determine the IHC scores. Histological scores were calculated based on “0—no inflammation, 1—mild, 2—moderate, 3—severe active”.

### Animals

IEC-specific conditional AhR knockout mice (AhR^ΔIEC^) were generated as described previously.^29^ Epithelial cells expressing EPCAM^+^ from the small intestine of wild-type (WT), and AhR^ΔIEC^ mice were isolated and analyzed for the mRNA and protein expression of AhR using RT-PCR and western blot analysis, respectively, to further confirm AhR cell-specific deletion in intestinal epithelial cells. C57BL/6, BALB/cJ, and B6.CB17-Prkdc^scid^/SzJ mice (8–10 weeks) were purchased from the Jackson Laboratory and housed in specific pathogen-free conditions, under 12-hour light/12-hour dark cycles in the Association for Assessment and Accreditation of Laboratory Animal Care – accredited (AAALAC-accredited) animal facility at the University of South Carolina School of Medicine. Mice were provided with unlimited access to water and fed a regular chow diet. All animal experiments and procedures were approved by the University of South Carolina Institutional Animal Care and Use Committee (IACUC) under the following protocol number: 2669-101803-070723.

### Induction of colitis, I3C treatment, and assessment of colitis parameters

Colitis in susceptible BALB/cJ mice was induced by intrarectal injections of TNBS (50 μL of 1 mg TNBS in 50% ethanol) under light isoflurane anesthesia, as previously reported.^6^ Mice in treatment groups received 100 μL i.p. injections of I3C (40 mg/kg in 0.05% DMSO/corn oil) 24 hours before TNBS injection and continued every day until completion of the experiment. Colitis in Prkdc^scid^/SzJ mice were induced by intraperitoneally (i.p.) injection of the anti-CD40, monoclonal antibody FGK45 (200 µg in PBS) as previous method.^64^ IgG2a (200 µg in PBS) was used as control. Colitis in C57BL/6 and AhR^ΔIEC^ mice was induced by water containing 3% DSS for 7 days, followed by a week of regular drinking water, as described previously.^6,65^ Mice in the treatment groups received 100 μL i.p. injections of I3C (40 mg/kg in 0.05% DMSO/corn oil) or VEH 24 hours before anti-CD40 administration or DSS, which were repeated every other day until the experiment was completed.^6^ For TCDD (provided by Dr. Steve Safe, Institute of Biosciences & Technology, Texas A&M Health Science Center, College Station, TX, United States) treatment, mice received a single dose of TCDD (25 µg/kg body weight in 100uL of corn oil) via i.p. injection 24 hours prior to DSS exposure or anti-CD40 treatment, as previously described.^66^ We collected blood, ileal contents, colon and ileal tissue samples at the end of the experiment. The colon was washed with phosphate-buffered saline and then sliced longitudinally, formalin-fixed, and paraffin-embedded. Ileal tissue control and experimental mice was used to isolate IECs as previously described.^67^

Colitis disease parameters such as body weight loss, colon lengths, and macroscopic colitis scores were assessed as described previously.^6,68,69^ The FITC-dextran assay was utilized to assess in vivo gut permeability as previously described by us previously.^6^ Briefly, mice with different models of colitis were given 600 mg/kg of 4kD FITC-dextran (Sigma-Aldrich) in 100 μL of PBS orally. Four hours later, blood from mice was collected, and a Biotek Synergy H4 multimode microplate reader set to 480 nm excitation wavelength was used to measure the quantities of FITC-dextran. Colonoscopy images were taken on day 3 of the TNBS model, day 9 of the Anti-CD40 model, and day 13 of the DSS model, using a Karl Storz Tele Pack Vet X LED endoscope. We evaluated the colonoscopy score as described previously using colonoscopy images.^70^ Histological scores in formalin-fixed colonic tissue sections stained with hematoxylin and eosin (H&E) were calculated using a combination of colonic tissue damage and inflammatory cell infiltration as criteria. The images were acquired using Discover ECHO Microscopes.

### Cell lines

We purchased mouse transgenic sertoli cell line 15P-1, IECs, MC38 (Murine adenocarcinoma), and Caco2 (Human colorectal adenocarcinoma) from ATCC, which uses PCR-based assays, karyotyping, and other methods to confirm the identity of cells. MC38 and 15P-1 were cultured in Dulbecco’s modified Eagle’s medium, whereas Caco2 cells were grown in an EMEM containing 10% FBS (Thermo Fisher Scientific) in a humidified atmosphere with 5% CO_2_ at 37°C.

### Antibodies, vectors, RNAi, and chemicals

AhR (Cat#sc-133088) was purchased from Santa Cruz Biotechnology (Santa Cruz, CA). AhR (Cat#MA1-514), and α-defensin 1 (Cat# PA5-103122) were obtained from Invitrogen (Rockford, IL). β-Actin (Cat#A5441) was procured from Sigma-Aldrich (St. Louis, MO). PE anti-mouse CD326 (Ep-CAM) antibody (Cat#118206), APC anti-mouse CD45.2 antibody (Cat#109814), mouse IgG2a (Cat#400202) were purchased from BioLegend (San Diego, CA). Normal rabbit IgG (Cat#2729) and HRP-conjugated anti-rabbit IgG (Cat#7074) were obtained from Cell Signaling Technology (Danvers, MA). HRP-conjugated anti-mouse IgG (Cat#W4028) was from Promega. Rat IgG2a isotype control (Cat# BE0089) and rat anti-CD40 recombinant antibody (clone FGK4.5) (Cat#BE0016-2) were received from BioXcell (Lebanon, NH). Goat anti-mouse IgG (H+L) highly cross-adsorbed secondary antibody, Alexa Fluor™ 488 (Cat# A-11029), goat anti-Mouse IgG (H+L) cross-adsorbed secondary antibody, Alexa Fluor™ 568 (Cat# A-11004), and goat anti-rabbit IgG (H+L) cross-adsorbed secondary antibody, Alexa Fluor™ 568 (Cat# A-11011) were purchased from Thermo Fisher Scientific.

pGL3-basic vector and renilla luciferase plasmids were obtained from Addgene. We purchased AhR siRNAs (Cat#sc-29654 (H) and sc-29655 (M)), control siRNA (catalog sc-37007), AhR shRNA (m) Lentiviral Particles (Cat#sc-29655-V) and Control shRNA Lentiviral Particles-A (Cat#sc-108080) from Santa Cruz Biotechnology. Indole-3-carbinol (I3C) (I7256-5G) was procured from Sigma-Aldrich. We purchased Lipofectamine 3000 (L3000-015), RNAiMAX (Cat#13778-15), halt™ protease inhibitor cocktail (100×) (Cat#78430), and RIPA Lysis and Extraction Buffer (Cat#89901) from Thermo Fisher Scientific. Dextran Sulfate Sodium (DSS) (Cat#160110) was from MP Biomedicals and TA Cloning™ Kit, with pCR™2.1 Vector and One Shot™ TOP10F’ Chemically Competent E. coli (Cat# K203001) was obtained from Thermo Fisher.

### Generation of the α-defensin 1 promoter reporter constructs

We amplified α-defensin1 promoter region containing DRE regions located ~9 kb upstream of the transcription start site (TSS) of α-defensin 1 using the below primers.

pGL3-α-D1-DRE1-F-5’- AAAAGCCTACGCTACCATG-3’

pGL3-α-D1-DRE1-R-5’- ATGTGTTAGGTGCAGCGTGT-3’

pGL3-α-D1-DRE2-F-5’- AACACACAACGCACACGCAC-3’

pGL3-α-D1-DRE2-R-5’- TCCCTTGCTTCAGGGAAGAT-3’

pGL3-α-D1-DRE3-F-5’- TCATCTTCCCTGAAGCAAGGG-3’

pGL3-α-D1-DRE3-R-5’- TACCAGCTCGACATGACAT-3’

pGL3-α-D1-DRE1+DRE2+DRE3-F-5’- AAAAGCCTACGCTACCATG-3’

pGL3-α-D1-DRE1+DRE2+DRE3-R-5’- TACCAGCTCGACATGACAT-3’

The resulting PCR products were directly into the TOPO-TA pCR™2.1 vector according to manufacturer’s instructions. The TOPO-TA vector’s α-defensin1 promoter regions were digested with KPNI and XhoI, then cloned into the pGL3-basic vector. These constructs were confirmed both by restriction digestion and DNA sequencing and designated as pGL3-α-D1-DRE1, pGL3-α-D1-DRE2, pGL3-α-D1-DRE3, and pGL3-α-D1-DRE1 + DRE2 + DRE3. We use α-D1-DREs to indicate all constructs.

### Reporter assays

pGL-3 α-D1-DREs and renilla plasmids were cotransfected with control or AhR siRNA into 15p-1 and MC38 cells. Renilla and firefly luciferase activities were assessed using the Dual-Luciferase kit (Promega, USA) according to the manufacturer’s guidelines. The transfection efficiency of luciferase assays was normalized using a renilla reporter vector. The results presented are indicative of three independent studies, each conducted in triplicate.

### Immunofluorescence, Western blotting, and RNA extraction and quantitative reverse-transcriptase PCR

Immunofluorescence and western blotting were performed as described previously.^29,63,65^ Cellular RNA was extracted using the Qiagen RNeasy Kit (Valencia, CA), and complementary DNA was synthesized using the Applied Biosystems high-capacity cDNA reverse transcription kit (Carlsbad, CA) in accordance with the manufacturer’s guidelines. qPCR was used to measure mRNA expression of AhR, α-defensin 1, EPCAM, and GAPDH in mouse and human samples using the primers listed below:

mAhR-F:5’-CTTCTAAGCGACACAGAGAC-3’

mAhR-R:5’-AATAACATCTTGCGGGAAGG-3’

α-defensin 1-F:5’- GGCCGTATCTGTCTCCTTTG-3’

α-defensin 1-R:5’- CTCTTCCTTTGCAGCCTCTT-3’

mGAPDH-F:5’- AACTTTGGCATTGTGGAAGG −3’

mGAPDH-R:5’- CAGGGATGATGTTCTGGGCA-3’

mEpCAM-F-5’-TTGCTCCAAACTGGCGTCTA-3’

mEpCAM-R-5’-ACGTGATCTCCGTGTCCTTGT-3’

hAhR-F:5’- CCATCCCCATACCCCACTAC −3’

hAhR-R:5’- TTCTGGCTGGCACTGATACA −3’

DEFA1-F:5’- CATCCTTGCTGCCATTCTCC −3’

DEFA1-R:5’- CCTGGTAGATGCAGGTTCCA −3’

hGAPDH-F:5’- GTCTCCTCTGACTTCAACAGCG −3’

hGAPDH-R:5’- ACCACCCTGTTGCTGTAGCCAA-3’

### Chromatin immunoprecipitation

We performed chromatin immunoprecipitation (ChIP) analysis using Pierce Magnetic ChIP kit (ThermoFisher Scientific, Waltham, MA) according to the manufacturer’s instructions to analyze the binding ability of AhR to the DRE of α-defensin1 promoter. Mouse nonspecific IgG was used as a control. qPCR was performed using the primers below to identify the interaction of AhR with the promoter of α-defensin 1. Primers for ChIP-qPCR was as follows:

α-D1-DRE3-F-5’- TCATCTTCCCTGAAGCAAGGG-3’

α-D1-DRE3-R-5’- TACCAGCTCGACATGACAT-3’

### Microbial 16S rRNA gene analysis

Microbial 16S rRNA sequencing analysis was performed using genomic DNA isolated from ileal contents of normal and colitis mice to identify the bacterial distribution as previously described.^6,71^ The Nephele platform from the National Institute of Allergy and Infectious Diseases (NIAID) Office of Cyber Infrastructure and Computational Biology (OCICB) in Bethesda, Maryland, USA, was used to analyze the sequenced data collected on the Illumina Miseq.^72^ Output files were processed using the Huttenhower group’s LefSe Galaxy online application tool to estimate gut microbial composition.^73^

### Whole-transcript (WT) expression arrays

Microarrays were performed as described previously.^74^ Briefly, total RNA was purified from ileal epithelial cells of control and experimental mice using the Qiagen RNA easy kit. After removal of DNA upon recombinant DNAse I treatment, Polyadenylated controls were prepared, and 1 mg of RNA was converted to complementary DNA (cDNA). These cDNAs were purified, quantified, and subsequently fragmented into 40–70 base pairs (bp). These cDNA fragments were labeled using the Affymetrix^Ⓡ^ genechip WT terminal labeling kit. The GeneChip™ Hybridization, Wash, and Stain kit was used with the Affymetrix® Hybridization Oven and Affymetrix® Fluidics Station on the 450 protocol to prepare Affymetrix® GeneChip® arrays. After hybridization, microarrays were washed in the Fluidics Station (Affymetrix Gene Chip Fluidics Station 450 FS; Affymetrix, Santa Clara, CA, USA) following the manufacturer’s instructions and scanned using the System Affymetrix Gene Chip Scanner 3000 7 G. Microarray intensity was quantified, and differential expression was analyzed using Applied Biosystems Transcription Analysis Console (TAC) 4.0 software. The heat maps were generated using the TAC 4.0 software. Expression data were analyzed using Ingenuity Pathway Analysis (IPA). GEO accession number for Whole-Transcript (WT) Expression Arrays is GSE285819.

## Statistical analysis

Statistical analysis was performed using GraphPad Prism 7. The immunohistochemical results were analyzed by a 2-tailed Student’s t-test. RT-PCR data were statistically analyzed by 1-way ANOVA followed by Tukey’s multiple comparison test. Results were shown as mean ± SEM. Results were considered significant if the *p* value was less than 0.05. In figures, *p < .05, **p < .01, ***p < .001, ****p < .001, NS = Not significant.

## Supplementary Material

Supplemental Material

## Data Availability

Whole-Transcript (WT) Expression Arrays are available on the Gene Expression Omnibus (GEO) Database (https://www.ncbi.nlm.nih.gov/geo/query/acc.cgi?acc=GSE285819). Further information and requests for resources and reagents should be directed to and will be fulfilled by the lead contact, Prakash Nagarkatti (prakash@mailbox.sc.edu).
