# Supplementary material for: AhR Activation Transcriptionally Induces Anti-Microbial Peptide Alpha-Defensin 1 Leading to Reversal of Gut Microbiota Dysbiosis and Colitis
Source: Gut Microbes. 2025 Feb 2;17(1):2460538. doi: 10.1080/19490976.2025.2460538 (PMC11792800; doi:10.1080/19490976.2025.2460538)

**Supplementary Fig. 1.** (A) IECs isolated from control and TNBS (A)-, Anti-CD40 (B)- and DSS (C)-induced colitis mice were identified by flow cytometric analysis using the cell surface marker EPCAM. (D) The same as C, but the treatment was TCDD. (E) The same as (D), but the colitis was induced by Anti-CD40.


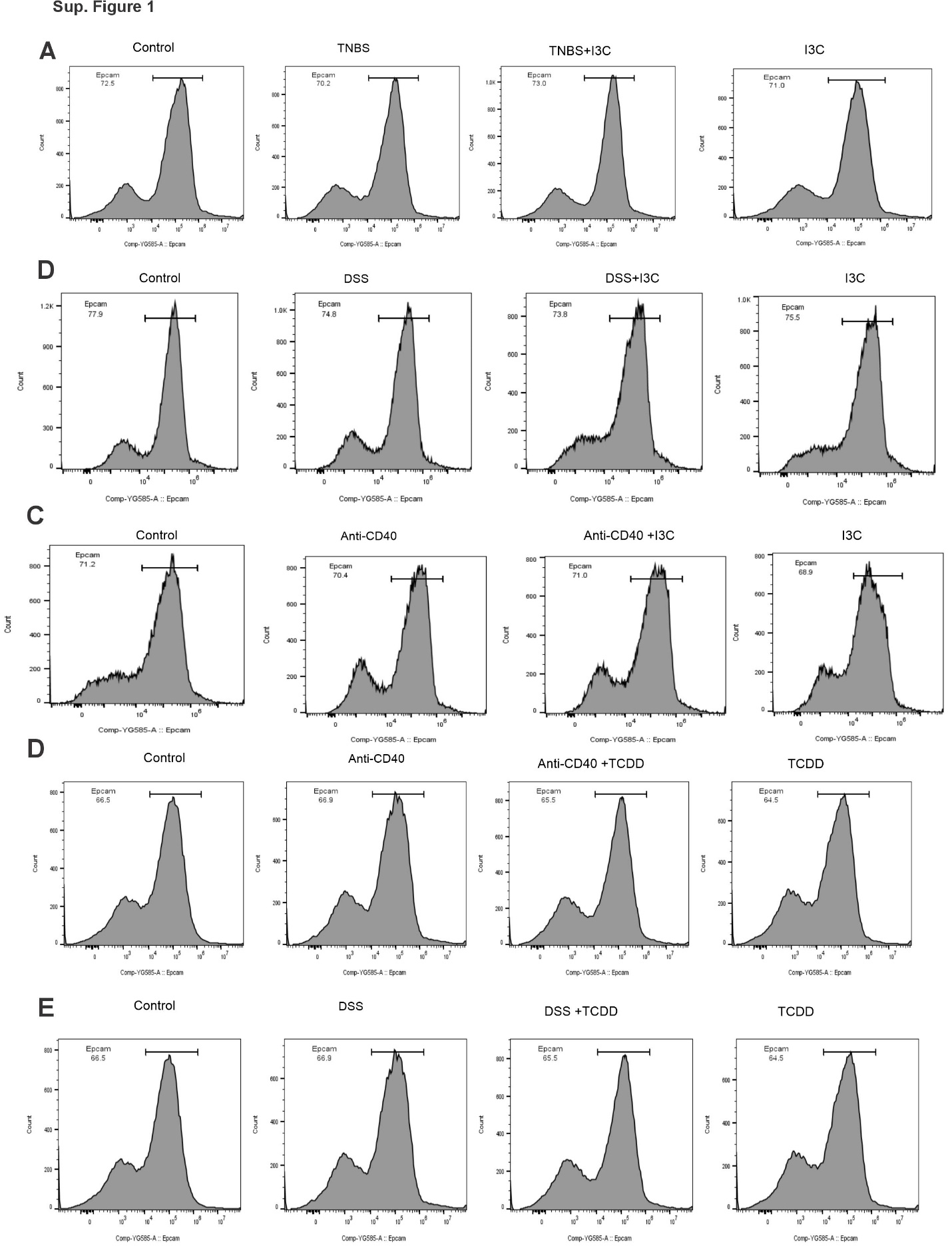


**Supplementary Fig. 2.** TCDD also enhances AhR and α-defensin 1 in IECs from control and DSS- and Anti-CD40-inudeced colitis mice. (A-C) AhR (A), α-defensin 1 (B) and CYP1A1 (C) mRNA expression in DSS-induced colitis model (n=6). (D) AhR, and α-defensin 1 protein expression in DSS-induced colitis model (n=6). (E-G) The same as A-C, but AHR (E) , α-defensin 1 (F) and CYP1A1 (G) mRNA and protein expression were analyzed in Anti-CD40-inudeced colitis mice model (n=6). (H) AHR, and α-defensin 1 protein expression were analyzed in Anti-CD40-inudeced colitis mice model (n=6). Bottom panel for D and H represents densitometry analysis of AhR and α-defensin 1 protein expression. Data are shown as mean ± SEM, and significance was determined using 1-way ANOVA and Tukey’s multiple comparisons test; **p < 0.01; ***p< 0.001. NS=Not significant.


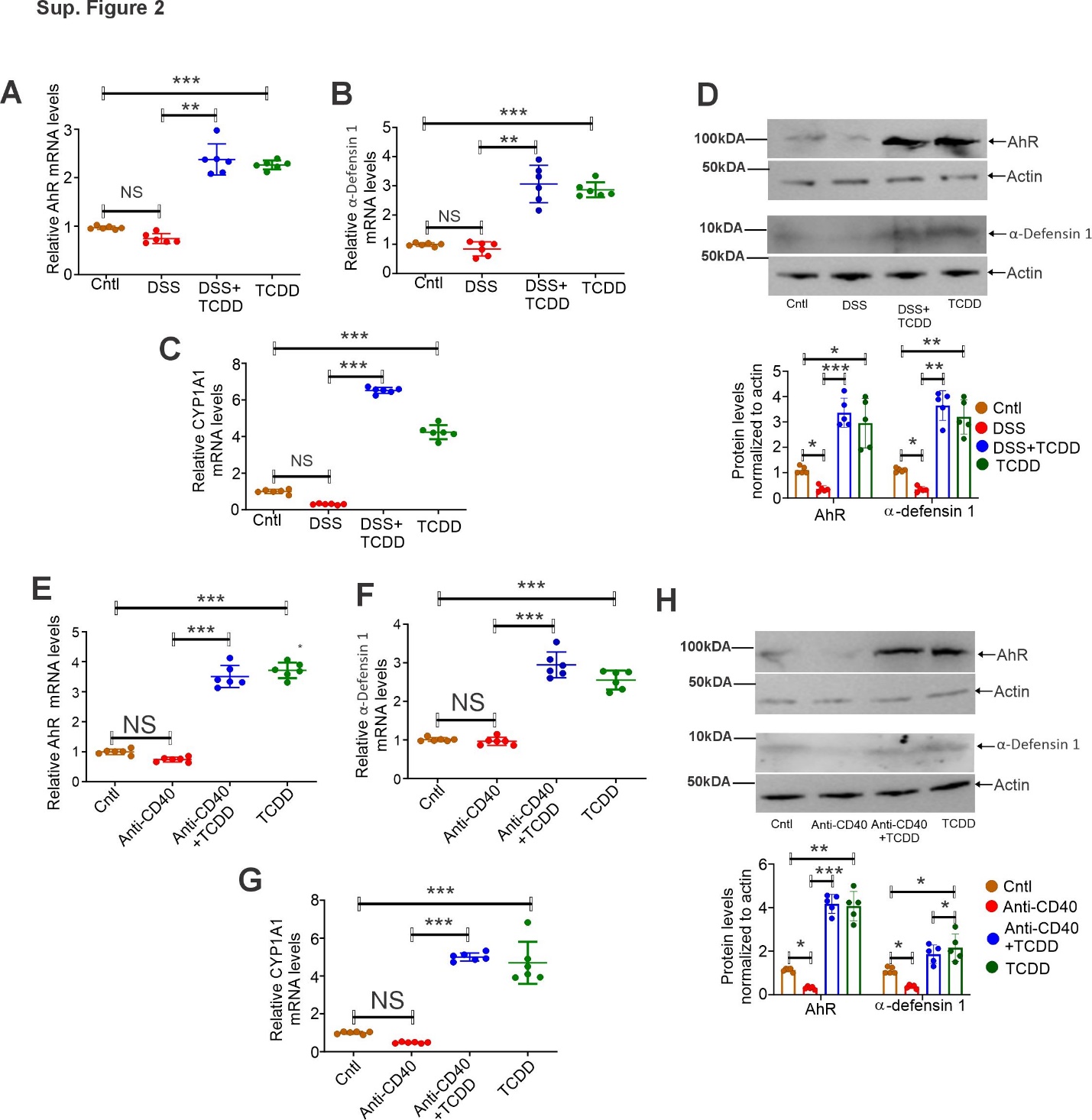


**Supplementary Fig. 3.**  Blocking AhR attenuates I3C-mediated upregulation of α-defensin 1 in IECs. (A) AhR and α-defensin 1 protein expression in 15p-1 cells was analyzed by western blotting (n=3). (B) Representative bar diagram depicts the quantification of immunofluorescence staining for AhR and α-defensin 1 protein expression (showed in figures 3C and 3D) in 15p-1 cells (n=3). (C) AhR and α-defensin 1 protein expression in MC38 cells was analyzed by western blotting (n=3). (D) Representative bar diagram depicts the quantification of immunofluorescence staining using ImageJ for AhR and α-defensin 1 protein expression in MC38 cells (n=3). (E, F) 15p-1 cells were pretreated with I3C, AhR antagonists, α-Naphthoflavone (NP; 5µM) or CH223191 (CH; 10µM) for 2hrs at indicated concentrations, and then treated with DSS (0.03%) for additional 16 hours. The mRNA expression of AhR (E) and α-defensin 1 (F) was analyzed by real-time PCR. (G, H) The same as A and B, but AhR (G) and α-defensin 1 (H) protein expression were analyzed by immunofluorescence staining. Bottom graph panel for G and H represents quantification of immunofluorescence staining using ImageJ for AhR and α-defensin 1 protein expression (n=3). Data are shown as mean ± SEM, and significance was determined using 1-way ANOVA and Tukey’s multiple comparisons test; *p < 0.05; ***p< 0.001. NS=Not significant.


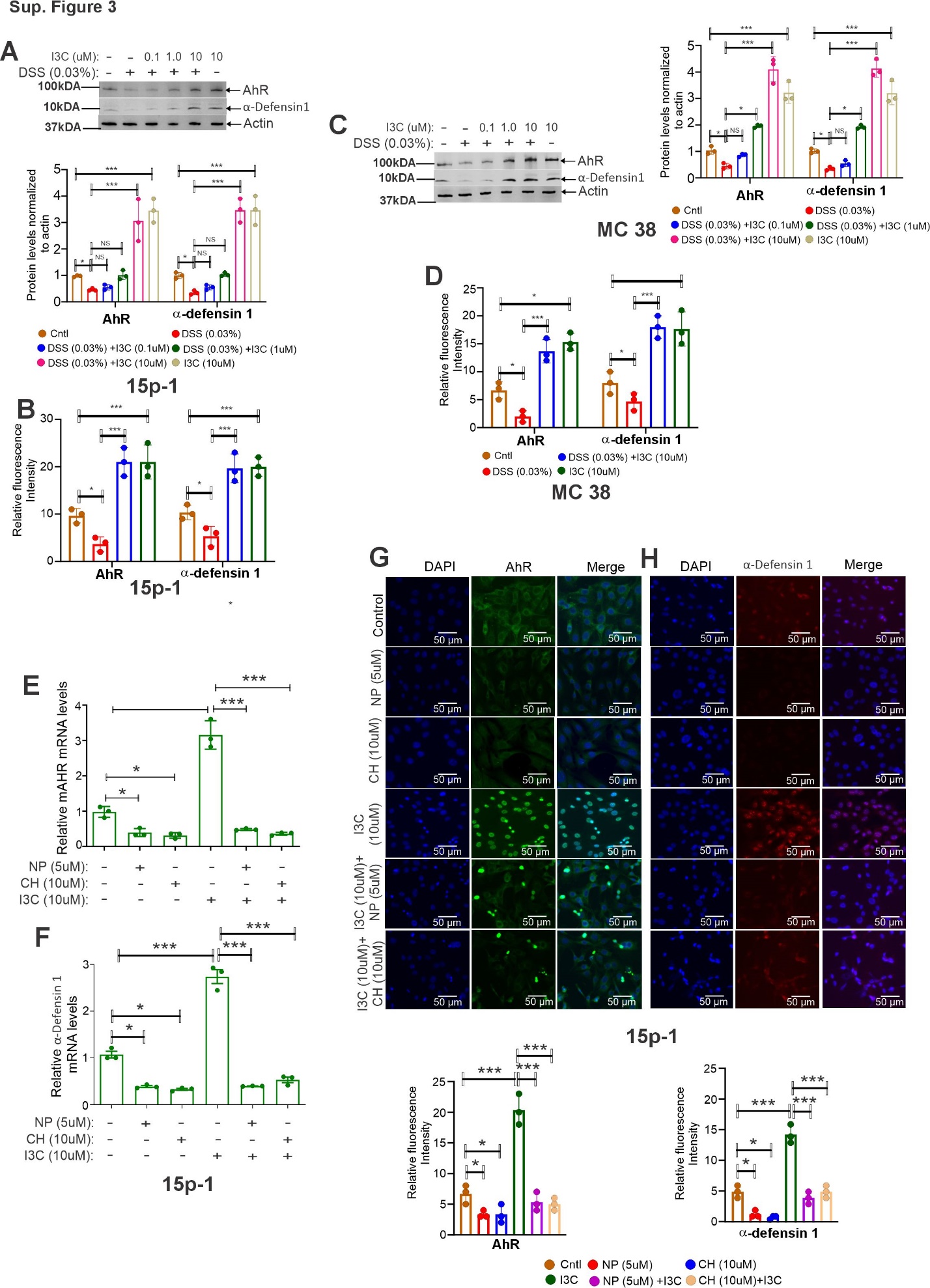


**Supplementary Fig. 4.**  (A) 15p-1 cells transiently transfected with AhR siRNA or control siRNA were pretreated with I3C, at indicated concentrations, and then treated with DSS (0.03%) for an additional 16 hours. AhR (A) and α-defensin 1 (B) protein expression was analyzed by western bloting (n=3). (B) Representative bar diagram depicts the quantification of immunofluorescence staining for AhR and α-defensin 1 protein expression using ImageJ in MC38 cells (n=3). (C) AhR and α-defensin 1 protein expression in MC38 cells was analyzed by western blotting (n=3). (D) Representative bar diagram depicts the quantification of immunofluorescence staining using ImageJ for AhR and α-defensin 1 protein expression in MC38 cells (n=3). (E) MC38 cells were co-transfected the pGL3-DRE1, pGL3-DRE2, pGL3-DRE3, and pGL3-DRE1+2+3 luciferase reporter plasmid with AhR siRNA or control siRNA and then treated with I3C and DSS at indicated concentration and luciferase reporter assays were performed (n=3). (F) The transfection efficiency of the reporter assay for AhR siRNA was measured via western blotting analysis with antibodies against AhR (upper panel) and RT-PCR analysis of AhR mRNA expression (bottom panel). All luciferase assays were normalized for transfection efficiency by renilla reporter activity. The results shown are a representative of four independent experiments performed each time in triplicate. Data are displayed as mean ± SEM. Significance was determined using 1-way ANOVA and Tukey’s multiple comparisons test; *p < 0.05; ****p* < 0.001, NS=Not significant.


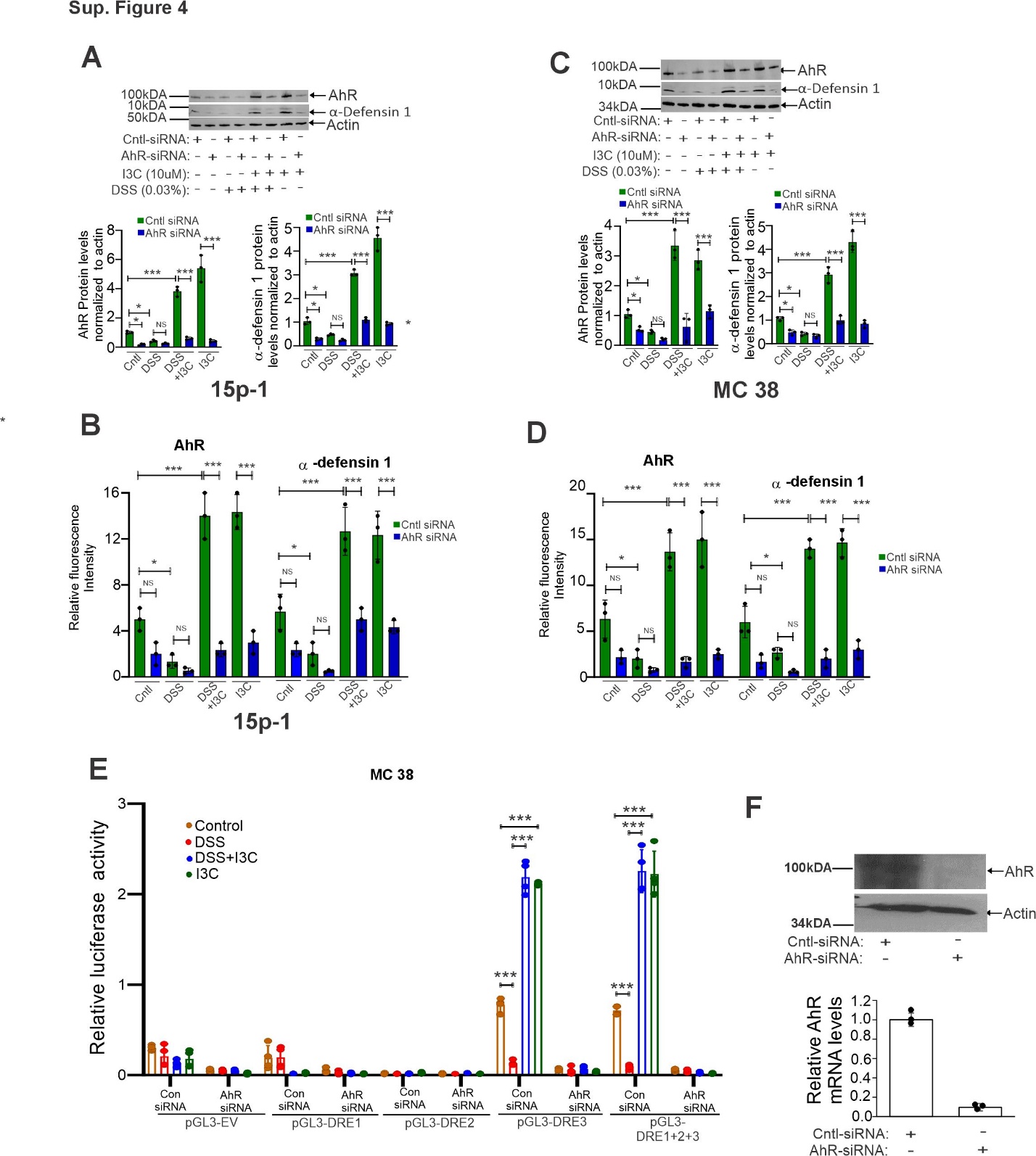


**Supplementary Fig. 5.**  AhR-α-defensin1 signaling inhibits DSS-induced colitis. (A) Experimental design for DSS-induced colitis in mice as described in Methods. (B–F) Colitis was assessed by percent weight loss (A), colon length (B), macroscopic score (C), serum FITC-dextran (E). (F) Representative colonoscopy images of the experimental and control animals. (G) Bar graph depicting colonoscopy scores from experimental mice. (H) Representative H&E stains of colons from experimental mice (*n* = 5). Scale bars: 150 μm (original magnification, ×10). (I) Bar graph depicting histopathological scores of H&E-stained colons from experimental mice (*n* = 6). Data are displayed as mean ± SEM. Significance was determined using 1-way ANOVA and Tukey’s multiple comparisons test; *p < 0.05; ***p < 0.001, NS=Not significant.


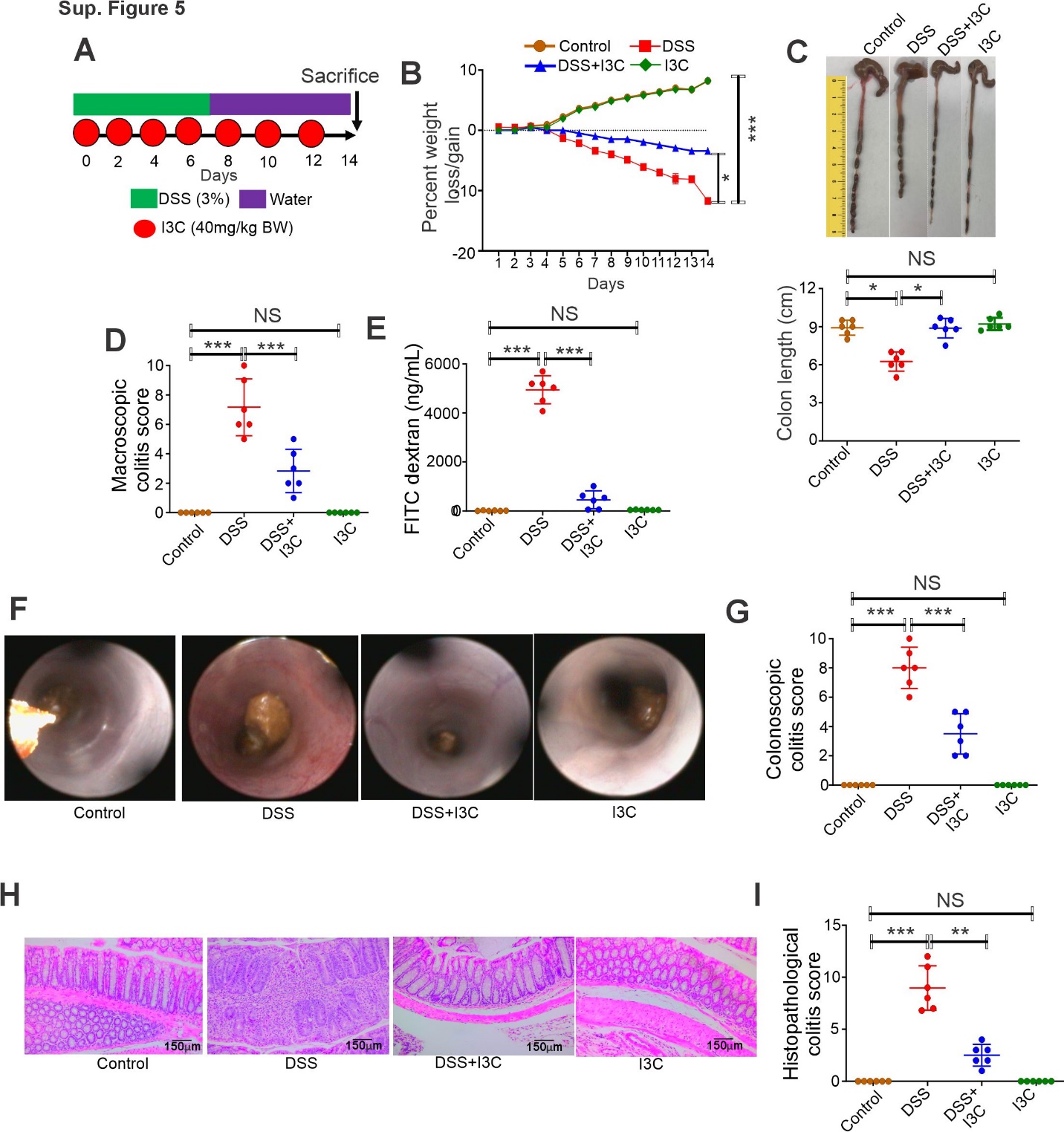


**Supplementary Fig. 6.**  AhR-α-defensin1 signaling inhibits TNBS-induced colitis. (A) Experimental design for TNBS-induced colitis in mice as described in Methods. (B–F) Colitis was assessed by percent weight loss (A), colon length (B), macroscopic score (C), serum FITC-dextran (E). (F) Representative colonoscopy images of the experimental and control animals. (G) Bar graph depicting colonoscopy scores from experimental mice. (H) Representative H&E stains of colons from experimental mice (*n* = 5). Scale bars: 150 μm (original magnification, ×10). (I) Bar graph depicting histopathological scores of H&E-stained colons from experimental mice (*n* = 6). Data are displayed as mean ± SEM. Significance was determined using 1-way ANOVA and Tukey’s multiple comparisons test; *p < 0.05; ***p < 0.001, NS=Not significant.


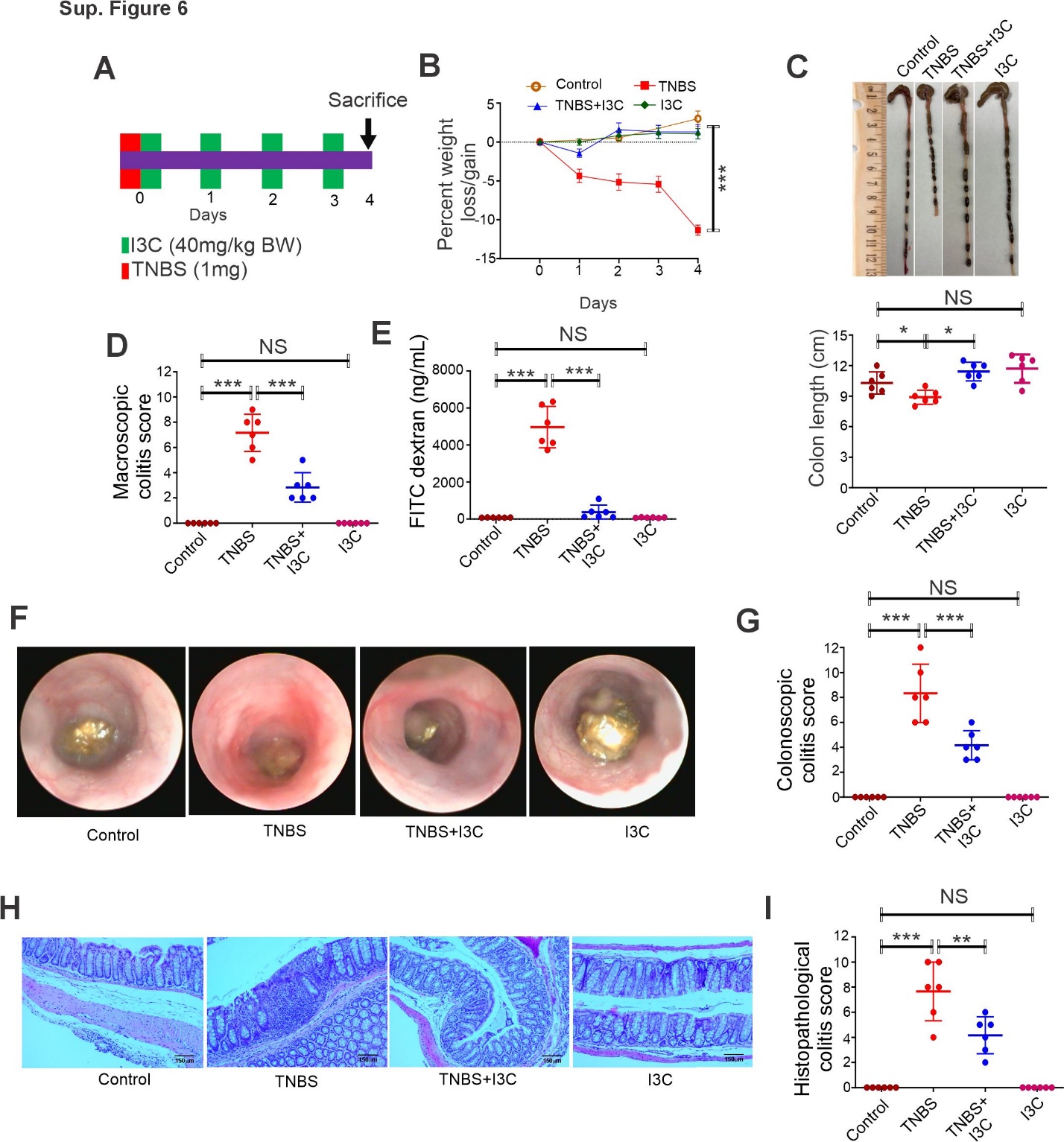


**Supplementary Fig. 7.** 16S rRNA sequencing was performed from the ileal contents of control and DSS-induced colitis. Sequenced reads were analyzed using Nephele to determine chao1 α diversity (A) and β diversity by PCA plot (B). (C, D) Cladogram depicting biomarkers between control vs DSS groups (C) and between DSS vs DSS+I3C groups (D). (E, F) LDA score depicting biomarkers between control vs DSS groups (C) and between DSS vs DSS+I3C groups (D). (G) A more detailed phylum distribution in control and experimental animals. The graph (right) panel shows the levels of Bacteroidota, Firmicutes and Verrucomicroiota phylum in control and colitis mice (n=4). (H) Heatmap depicts a more detailed species distribution, and the graph panel (I) represents the levels of various species in control and experimental animals (n=4). Data are shown as mean ± SD; n=4. Significance was determined using 1-way ANOVA and Tukey’s multiple comparisons test; *p < 0.05; ****p < 0.001, NS=Not significant.


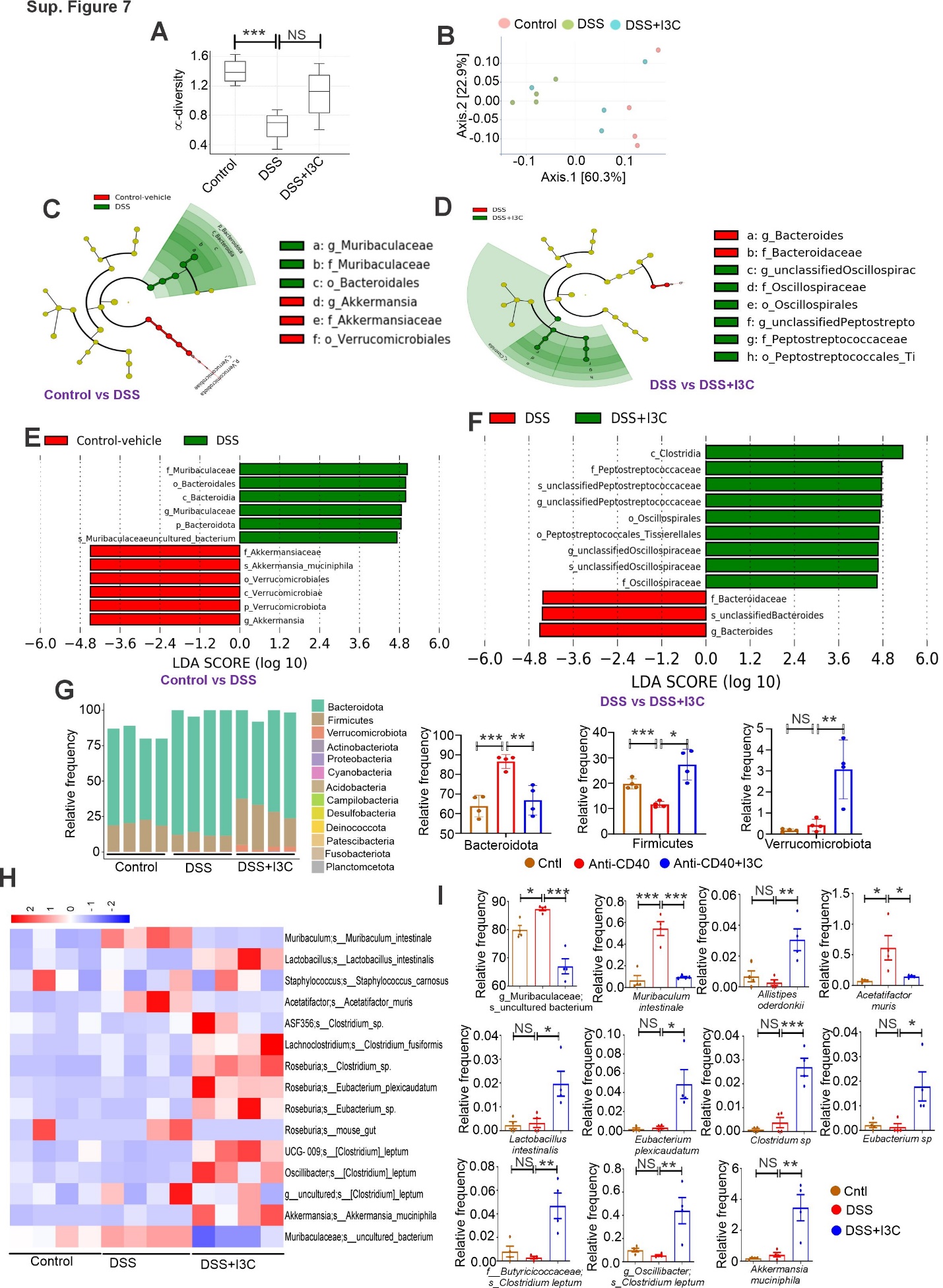

Supplement: Supplemental Material [file KGMI_A_2460538_SM0360.docx]
